# Treatment patterns in people with cystic fibrosis: have they changed since the introduction of ivacaftor?

**DOI:** 10.1016/j.jcf.2021.08.014

**Published:** 2022-03

**Authors:** Emily Granger, Gwyneth Davies, Ruth H. Keogh

**Affiliations:** a Department of Medical Statistics, Faculty of Epidemiology and Population Health, London School of Hygiene and Tropical Medicine, Keppel St, Bloomsbury, London WC1E 7HT, United Kingdom; b Population, Policy and Practice Research and Teaching Department, UCL Great Ormond Street Institute of Child Health (UCL GOS ICH), London WC1N 1EH, United Kingdom; c Centre for Statistical Methodology, London School of Hygiene and Tropical Medicine, Keppel St, Bloomsbury, London WC1E 7HT, United Kingdom

**Keywords:** cystic fibrosis, ivacaftor, treatment patterns, treatment burden, registry data

## Abstract

**Background:**

In late 2012, ivacaftor became available in the UK for people with cystic fibrosis (CF) aged 6 years and over with a G551D mutation. Long-term changes in treatment patterns have not previously been reported. We investigated long-term treatment patterns in people with CF with a G551D mutation who took ivacaftor and compared these with non-ivacaftor-treated cohorts using the UK Cystic Fibrosis Registry.

**Methods:**

Using 2007-2018 data we compared treatment patterns between four cohorts: 1: ivacaftor-treated; 2: ivacaftor era (2013-2018), ineligible genotype (no G551D mutation); 3: pre-ivacaftor era (2007-2012), eligible genotype (G551D mutation); 4: pre-ivacaftor era, ineligible genotype. Treatments included: inhaled antibiotics, dornase alfa, hypertonic saline, chronic oral antibiotics and supplementary feeding.

**Results:**

Up to 2012 the percentages of people taking each treatment were similar between the two cohorts defined by genotype and tended to increase by year with a similar slope. Once ivacaftor was introduced, the use of other treatments tended to decrease or remain stable by year for the ivacaftor-treated cohort, whereas it remained stable or increased in the non-ivacaftor-treated cohort. This led to differences in treatment use between the two cohorts in the ivacaftor-era, which became more marked over time.

**Conclusions:**

We have shown a clear divergence in treatment patterns since the introduction of ivacaftor in a number of key treatments widely used in CF. Further research is needed to investigate whether the differences in treatment patterns are associated with changes in health outcomes.

## Highlights

• Longitudinal treatment patterns among the ivacaftor-treated cystic fibrosis population in the UK differ from those seen in a contemporary cohort of individuals untreated due to their genotype, despite minimal differences between the genotype groups prior to the introduction of ivacaftor.
• People who are treated with ivacaftor were less likely to continue other treatments such as inhaled antibiotics, dornase alfa, hypertonic saline, chronic oral antibiotics and supplementary feeding, compared to people who are not treated with ivacaftor.
• The differences in use of dornase alfa and hypertonic saline solution between ivacaftor-treated and non-ivacaftor-treated people, are larger for people with higher lung function.

## 1 Introduction

Ivacaftor was the first CF transmembrane conductance regulator (CFTR) modulator therapy to be licensed for the treatment of people with cystic fibrosis (CF) with specific CF-causing genetic mutations. Phase III randomized controlled trials have found evidence that treatment with ivacaftor is associated with significant improvement in clinical outcomes including FEV_1_ for individuals with a Gly551Asp-CFTR (G551D) mutation [1,2]. Ivacaftor has been prescribed as standard care in the UK to people with CF aged 6 years and over with a G551D mutation since 2013, with access later expanded to individuals with eight other gating mutations, aged six months and older, and with an Arg117His-CFTR mutation. Recent studies of the effect of ivacaftor in the eligible CF population have been undertaken using registry data from the UK and US, and have found that treatment with ivacaftor is associated with improved outcomes including better preserved lung function, improved nutritional status and decreased risk of hospitalisations [3], [4], [5], [6].

Treatment burden is an important factor in the quality of life of people with CF and identifying ways to reduce treatment burden is a top research priority for the CF community [7,8]. The effect of ivacaftor use on patient reported outcomes was studied in a randomized controlled trial using the Cystic Fibrosis Questionnaire-Revised (CFQ-R) by Quittner et al. [9], who found evidence of reduced treatment burden in the ivacaftor-treated group, though this did not capture the nature of any reduction in the treatment burden. Reasons may include changes in perception of ‘burden’ following introduction of modulators, or withdrawal of treatments following modulator introduction. To our knowledge, long-term changes in treatment patterns in ivacaftor-treated patients have not been reported to date. Volkova et al. reported prevalence of treatments such as dornase alfa, hypertonic saline and chronic antibiotics at the time of initiating ivacaftor in the UK and US patient registries, but not at follow-up [6]. Hubert et al. reported findings from a retrospective French multi-centre study 1-2 years after initiating ivacaftor, including an observation that the proportion of patients taking chronic therapies such as nebulised dornase alfa decreased over this period [10].

We hypothesised that the introduction of ivacaftor would lead to changes in the long-term use of other key treatments in CF and aimed to investigate this in a large observational dataset. In this study we use UK CF Registry data to describe treatment use up to 6 years after initiating ivacaftor in individuals with a G551D mutation. The documentation of all chronic treatments in the registry permits an opportunity to evaluate these in detail. Treatments considered are inhaled antibiotics, chronic oral antibiotics, dornase alfa, hypertonic saline and supplementary feeding.  To appropriately investigate associations between ivacaftor and other long-term treatment use, methodology should also take account temporal changes in treatment use in the wider CF population. We therefore compare treatment patterns in ivacaftor-treated individuals with patterns in individuals in the same time period but not receiving ivacaftor due to their genotype. We also compare treatment patterns over time in similar cohorts defined by genotype, but observed in the time period before ivacaftor became available. The clinical characteristics of the cohort defined by genotype and time period are summarised, and we investigate whether any differences in treatment patterns between groups differs between FEV_1_%, age, and sex.

## 2 Methods

### 2.1 Data source

We used data from the UK Cystic Fibrosis Registry, a national database sponsored and managed by the Cystic Fibrosis Trust. The registry was established in 1995 and records demographic data and annual review data on clinical measurements and treatment use, on nearly all people with CF in the UK [11]. National Health Service (NHS) Research Ethics approval has been granted for data collection into the registry and each patient, or their parent/guardian, provides written consent.

Ivacaftor was introduced in the UK in 2013. For this study we used registry data from 2007-2018, giving six years before ivacaftor was introduced (pre-ivacaftor era: 2007-2012) and six years after (ivacaftor era: 2013-2018) to compare longitudinal patterns of treatment use in the pre-ivacaftor and ivacaftor eras. The following treatments were considered: inhaled antibiotics, chronic oral antibiotics, dornase alfa, hypertonic saline and supplementary feeding. For chronic oral antibiotics we considered all types combined, and also azithromycin and flucloxacillin separately due to the different clinical indications for these being prescribed for long-term use. Supplementary feeding was considered separately according to whether it was oral or by gastrostomy. Use of ivacaftor and each other treatment (yes/no) over the past year is available for each annual review recorded in the registry. Dates of starting and stopping ivacaftor were also available and were used to verify ivacaftor use for all individuals and identify individuals who stopped ivacaftor during the follow-up period.

We also used registry data on genotype, age, sex, FEV_1_% predicted (recorded at each annual review, measured using the GLI equations [12]), and number of days intravenous (IV) antibiotic use over the past year (recorded at each annual review).

### 2.2 Statistical analysis

We investigated patterns of treatment use over the 6 years in the two eras (2007-2012, 2013-2018) both in ivacaftor-treated individuals (who started using ivacaftor in the ivacaftor era) and in individuals who were not eligible for ivacaftor due to their genotype. This enables an investigation of whether any changes in treatment patterns seen in the ivacaftor users in the ivacaftor era could be due in part to general changes over time in treatment use.

Ivacaftor was first licenced in the UK for people aged 6 and older with a G551D mutation in late 2012 (England) and 2013 (Scotland, Northern Ireland), and later licenced for individuals with other gating mutations (2015) or the R117H mutation (2018), and younger patients. We focus on individuals with a G551D mutation who began taking ivacaftor in 2012 or 2013; these patients form the ‘ivacaftor-treated’ cohort, and the majority (92.2%) initiated ivacaftor in 2013. We did not include people who started ivacaftor in later years because we are interested in patterns of other treatments as a function of the time since starting ivacaftor. For this study an ‘eligible genotype’ was defined as having at least one G551D mutation.

We defined four cohorts of individuals: ivacaftor-treated (2013-2018) (IVA-ELIG); ivacaftor era (2013-2018), ineligible genotype (IVA-INELIG); pre-ivacaftor era (2007-2012), eligible genotype (PRE-IVA-ELIG); pre-ivacaftor era (2007-2012), ineligible genotype (PRE-IVA-INELIG).

The ‘baseline year’ was defined as the year 2013 for cohorts IVA-ELIG and IVA-INELIG (ivacaftor era), and 2007 for cohorts PRE-IVA-ELIG and PRE-IVA-INELIG (pre-ivacaftor era). For all cohorts, we excluded children who were under 6 years old at baseline and people who received a transplant (from the year in which the transplant was recorded onwards). We excluded people from IVA-ELIG if they stopped treatment with ivacaftor (from the year they stopped onwards) and from IVA-INELIG if they started ivacaftor during a later year (from the year ivacaftor treatment initiated onwards), when it became available for individuals with non-G551D mutations. Many individuals appear in both IVA-ELIG and PRE-IVA-ELIG or in IVA-INELIG and PRE-IVA-INELIG as they have data recorded in the registry in both time periods.

There is some missing data on use of chronic treatments and we used a pragmatic imputation approach to address this. Where a missing value appeared in a year between two non-missing values that were the same, we set the missing value to be the same as the two non-missing values. For example, if a person was missing data on use of a given treatment in 2014, but was recorded as taking this treatment in 2013 and 2015, we assumed they were also taking it in 2014. After this procedure, observations with remaining missing data were excluded for the year the data were missing. To assess the sensitivity of results to this imputation method, we repeated the analyses twice using different methods: all missing data were imputed as either 0 (indicating no treatment use) or 1 (indicating treatment use).

We summarised key characteristics of individuals in the four cohorts by year (age, FEV_1_% and annual number of days on intravenous therapy (IV) (including hospital admissions and home courses)). For each cohort, we calculated the proportions of people taking each of the treatments of interest by year, and corresponding 95% confidence intervals. Results are presented graphically to illustrate changes in treatment use over time in the four groups. The analysis looking at proportions of users over time was repeated separately in males and females, in children and adults (using age at baseline year), and by FEV_1_% in the baseline year (FEV_1_%≤60, 60<FEV_1_%≤80, FEV_1_%>80). Chi-squared tests were used to test for differences in treatment use in the eligible and ineligible genotype groups in the baseline year and in the final year in the pre-ivacaftor era and the ivacaftor era. Hypothesis tests were not conducted for all years to avoid issues with multiple testing. We also investigated whether the trend in the proportions of treatment use over time differs by genotype group, in the pre-ivacaftor era and the ivacaftor era, separately. This analysis used a group-level logistic regression of the proportions of treatment use on genotype group, time, and their interaction. A test of the null hypothesis that the interaction term is equal to zero corresponds to a test of whether there is a difference in the trend of proportions over time by genotype group. All analyses were conducted in R version 3.6.1.

## 3 Results

Table 1 presents the number of people in each cohort by year, along with descriptive statistics. Among those with the eligible genotype, there were 402 individuals in the ivacaftor-treated cohort (IVA-ELIG)) and 287 in the pre-ivacaftor era (PRE-IVA-ELIG) in the baseline year. Among those with an ineligible genotype, there were 6588 individuals in the ivacaftor era (IVA-INELIG) and 3874 in the post-ivacaftor era (PRE-IVA-INELIG) in the baseline year. The numbers in each cohort may increase by year, if new people enter the registry who meet the inclusion criteria, or decrease, if existing people are excluded or lost to follow-up. A flowchart showing patient selection for each cohort by year is provided in the supplementary material (Supplementary Figure 1). The total numbers of people in cohorts IVA-ELIG, IVA-INELIG, PRE-IVA-ELIG and PRE-IVA-INELIG at any time point were 416, 7347, 429 and 6236, respectively. There is no entry to the ivacaftor-treated cohort after the baseline year, whereas individuals can enter as well as exit the other three cohorts. The analyses were repeated restricting to individuals observed in the baseline year and results were similar to those reported here.

**Table 1:** Summary of the numbers of people in each group by year and their characteristics (mean age; mean percent forced expiratory volume per 1 second (FEV_1_%); number (and proportion) of intravenous therapy (IV) days over the course of the year); number (and proportion) of people with pancreatic insufficiency (PI).

		2007	2008	2009	2010	2011	2012	2013	2014	2015	2016	2017	2018
		*Pre ivacaftor era, eligible genotype (PRE-IVA-ELIG)*						*Ivacaftor treated (IVA-ELIG)*					
N		287	273	344	354	374	360	402	394	388	382	375	357
Age		20.3	21.5	22.3	22.8	23.9	25.1	22.2	23.2	24.1	24.9	25.7	26.0
FEV1%		72.2	71.9	69.4	67.3	68.5	66.2	73.8	75.5	75.6	74.2	73.1	73.3
IV=0		154 (53.7%)	156 (49.1%)	206 (50.0%)	216 (47.2%)	220 (45.2%)	218 (45.3%)	203 (50.5%)	259 (65.7%)	258 (66.5%)	256 (67.0%)	244 (65.1%)	232 (65.0%)
IV:1-14		53 (18.1%)	49 (17.2%)	68 (16.9%)	77 (18.6%)	67 (13.6%)	74 (15.8%)	69 (17.2%)	61 (15.5%)	50 (12.9%)	56 (14.7%)	53 (14.1%)	54 (15.1%)
IV:15-28		26 (9.1%)	37 (12.5%)	42 (11.9%)	46 (12.1%)	60 (13.9%)	56 (13.1%)	48 (11.9%)	25 (6.3%)	31 (8.0%)	30 (7.9%)	33 (8.8%)	27 (7.6%)
IV>28		56 (19.2%)	60 (21.2%)	77 (21.2%)	84 (22.0%)	112 (27.3%)	104 (25.8%)	82 (20.4%)	49 (12.4%)	49 (12.6%)	40 (10.5%)	45 (12.0%)	44 (12.3%)
PI		189 (90.4%)	224 (91.1%)	289 (90.9%)	312 (91.8%)	329 (90.9%)	320 (89.6%)	365 (91.2%)	357 (91.5%)	353 (92.2%)	349 (91.6%)	340 (92.4%)	319 (89.9%)

		*Pre ivacaftor era, ineligible genotype (PRE-IVA-INELIG)*						*Ivacaftor era, ineligible genotype (IVA-INELIG)*					
N		3874	4321	5028	5219	5488	5410	6588	6675	6608	6495	6370	6141
Age		21.2	21.9	22.6	23.4	24.5	25.4	22.9	24.1	25.0	25.8	26.7	27.5
FEV1%		70.4	69.7	68.7	67.9	67.8	66.3	69.9	69.6	69.0	68.6	66.3	66.0
IV=0		2094 (54.1%)	2150 (49.8%)	2377 (47.3%)	2364 (45.3%)	2392 (43.6%)	2387 (44.1%)	3088 (46.9%)	3108 (46.6%)	3175 (48.0%)	3053 (47.0%)	3012 (47.3%)	2825 (46.0%)
IV:1-14		602 (15.5%)	715 (16.5%)	815 (16.2%)	863 (16.5%)	890 (16.2%)	844 (15.6%)	1079 (16.4%)	1058 (15.9%)	1058 (16.0%)	1057 (16.3%)	1052 (16.5%)	1025 (16.7%)
IV:15-28		377 (9.7%)	446 (10.3%)	599 (11.9%)	583 (11.2%)	663 (12.1%)	647 (12.0%)	746 (11.3%)	810 (12.1%)	748 (11.3%)	750 (11.5%)	721 (11.3%)	768 (12.5%)
IV>28		801 (20.7%)	1010 (23.4%)	1237 (24.6%)	1409 (27.0%)	1543 (28.1%)	1532 (28.3%)	1675 (25.4%)	1699 (25.5%)	1627 (24.6%)	1635 (25.2%)	1585 (24.9%)	1523 (24.8%)
PI		2754 (90.6%)	3514 (90.1%)	4142 (89.4%)	4484 (89.0%)	4743 (88.0%)	4617 (86.9%)	5621 (86.6%)	5691 (86.0%)	5588 (85.5%)	5478 (86.1%)	5347 (85.7%)	5214 (85.2%)

Average ages for the four cohorts ranged between 20.3 and 22.9 at baseline and increase by year. The average FEV_1_% at baseline was highest in the ivacaftor-treated cohort at 73.8 (measured in the first year that they started ivacaftor). This increased to 75.6 in 2015 and back down to 73.3 in 2018. For the remaining cohorts, the average FEV_1_% tended to decrease over time. The proportions of people in each category of total IV days were similar across cohorts in the baseline year, but trends over time differed. For example, the proportion of people with zero IV days increased over time in the ivacaftor-treated cohort, but decreased for the other three cohorts. The proportions with pancreatic insufficiency at baseline ranged between 86.6% (IVA-INELIG) and 91.2% (IVA-ELIG). In the ivacaftor-era, there were consistently higher proportions of people with pancreatic insufficiency in the ivacaftor-treated cohort, compared to the ineligible cohort.

**Fig. 1:**
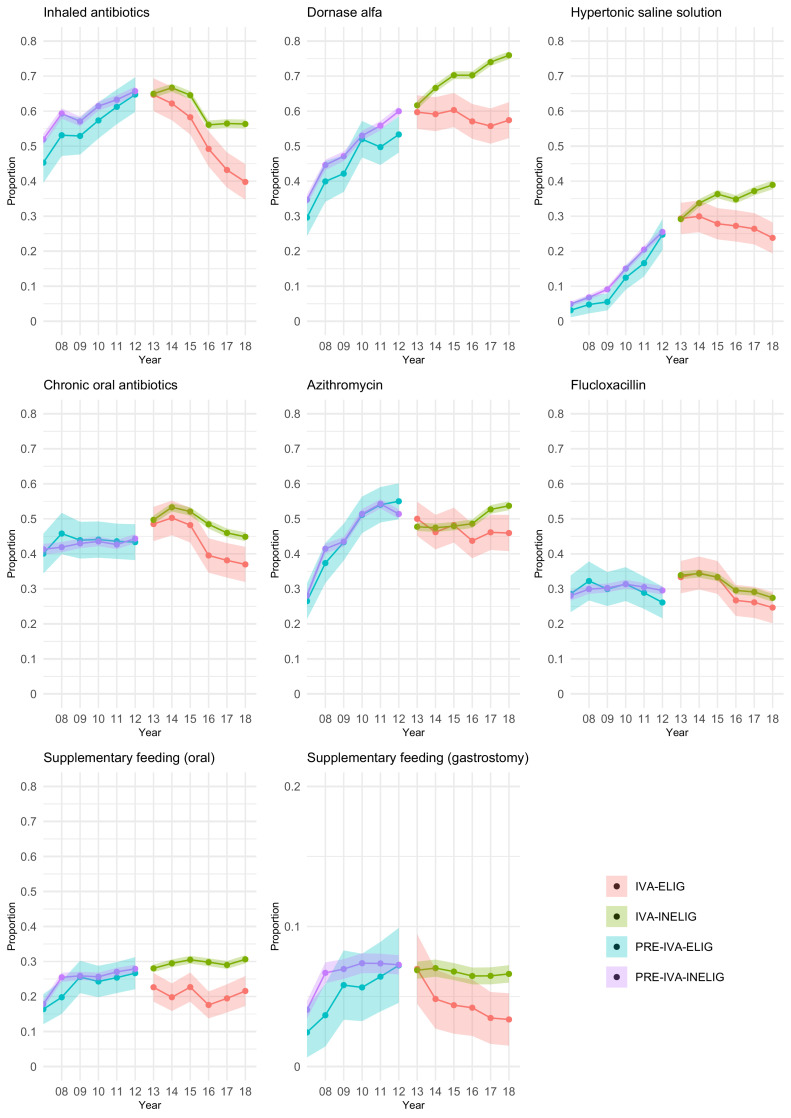
The proportions and 95% confidence intervals in each cohort prescribed different treatments by year.

Figure 1 presents the proportions (and 95% confidence intervals) in each cohort prescribed different treatments by year. Corresponding numerical results are shown in Supplementary Table 1. For all treatments, in the pre-ivacaftor era, the proportions are similar between the two genotype cohorts in 2007 and tend to increase similarly in both cohorts until 2012. For example, in 2007 45% (95% CI: 40-51%) of the eligible genotype group and 52% (50-54%) of the ineligible genotype group used inhaled antibiotics, and at the end of the pre-ivacaftor era in 2012 65% (60-70%) of the eligible genotype group and 66% (64-67%) of the ineligible genotype group used inhaled antibiotics. Hypothesis tests indicate no evidence of differences in treatment use between genotype groups in 2007 or 2012, except for inhaled antibiotics in 2007 (p=0.035) and dornase alfa in 2012 (p=0.015) (Supplementary Table 1). After ivacaftor was introduced, we observe differences in treatment patterns in the two genotype groups. For inhaled antibiotics and chronic oral antibiotics, treatment use decreases over time for both genotype groups in the ivacaftor era, but more so in the ivacaftor-treated cohort. For example, in 2013 65% of individuals had used inhaled antibiotics in both genotype groups (60-69% for IVA-ELIG and 64-66% for IVA-INELIG), while in 2018 this had decreased to 40% (35-45%) in the eligible genotype group (ivacaftor users) compared to 56% (55-58%) in the ineligible genotype group. For dornase alfa, hypertonic saline solution and azithromycin, treatment use continues to increase in the ivacaftor era for the ineligible genotype cohort, but decreases for the ivacaftor-treated cohort. Proportions using supplementary feeding (oral or gastrostomy) are generally much lower across all cohorts (with particularly low proportions observed for gastrostomy, hence the y-axis for this subplot is on a different scale). In the ivacaftor era the proportions using supplementary feeding remain approximately flat over time in both genotype cohorts for supplementary feeding, though with the proportions being lower in the ivacaftor-treated cohort. Hypothesis tests indicate significant differences in treatment use between genotype cohorts in 2018, for all treatments except flucloxacillin (Supplementary Table 1). Flucloxacillin was the only treatment for which treatment patterns were similar between the genotype groups across all years.

**Fig. 2:**
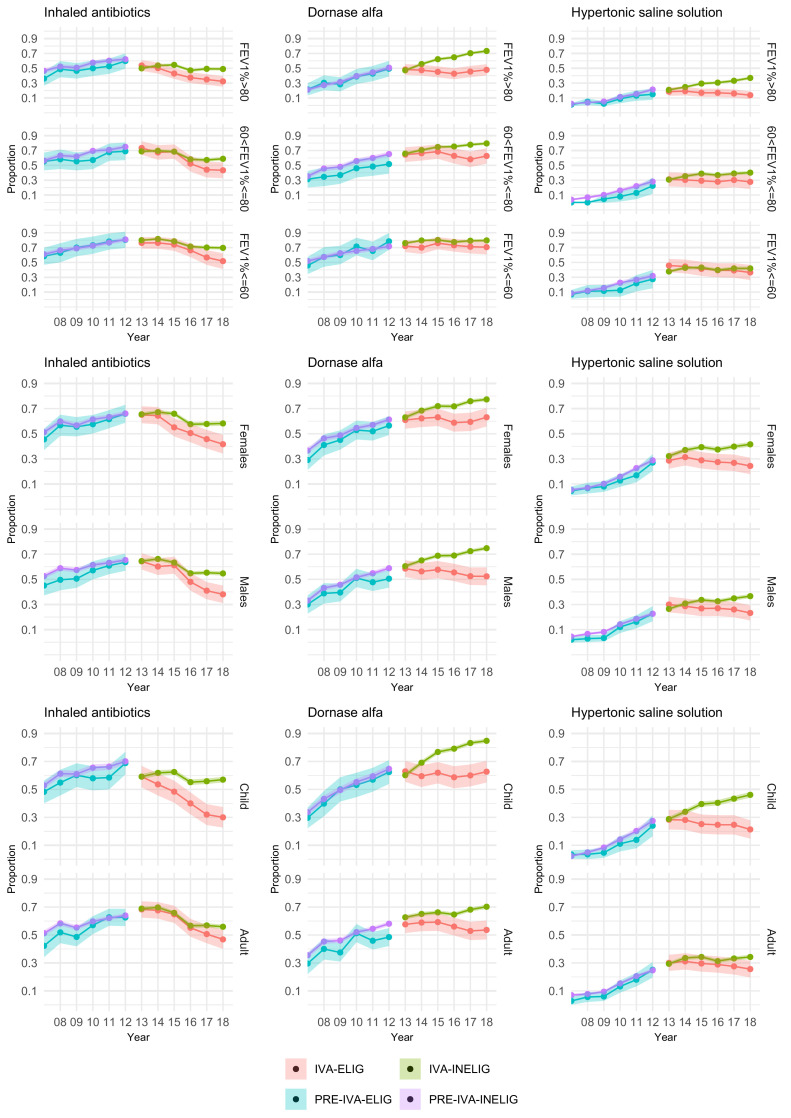
The proportions and 95% confidence intervals in each cohort prescribed inhaled antibiotics, dornase alfa and hypertonic saline solution by year, stratified by FEV_1_% at baseline, sex and age.

Figure 2 presents results separately by FEV_1_% in the baseline year, sex and age. Results are shown for inhaled antibiotics, dornase alfa and hypertonic saline solution. Further results are given in Supplementary Tables 2-8 and Supplementary Figures 2-4.

In the pre-ivacaftor era treatment use is similar in both genotype groups across all three FEV_1_% subgroups. For inhaled antibiotics, dornase alfa and hypertonic saline, differences in treatment use between the two genotype groups at the end of the ivacaftor era are larger for people with high lung function (FEV_1_%>80), compared to those with moderate or low lung function. Lower proportion of patients prescribed dornase alfa and hypertonic saline were also noted in children, with the close correlation between age and FEV_1_% noted. The subgroup analyses are described further in the online supplementary data.

We found no evidence of a difference in the trend of proportions of treatment use over time between the two genotype groups in the pre-ivacaftor era (Supplementary Table 9). In the ivacaftor-era, there was a significant difference (at the 5% significance level) in the trend of proportions over time between genotype groups, for all treatments except flucloxacillin and oral supplementary feeding (Supplementary Table 9).

Results were very similar between different missing data imputation methods, indicating that the observed trends were not sensitive to how missing data were handled (results not shown).

## 4 Discussion

We have shown a clear divergence in treatment use over time between individuals treated with ivacaftor and those untreated due to their genotype for a number of long-term treatments widely used in CF, including dornase alfa, hypertonic saline and inhaled antibiotics. When comparing treatment patterns in the two genotype groups in the time period before the introduction of ivacaftor we saw few differences, suggesting that the differences in treatment use seen in the ivacaftor era are not explained by other differences between the two genotype groups. We found evidence for treatment differences between ivacaftor-treated and comparator groups for dornase alfa and hypertonic saline within two years of ivacaftor initiation. Differences in treatment use between the ivacaftor-treated and comparator group were most pronounced for children and for those with high lung function (FEV_1_%>80). Our analysis represents the first detailed description of treatment patterns within the UK ivacaftor-treated population with a G551D mutation following its introduction into routine clinical care. Hubert et al. [7] found a decrease in prescribed dornase alfa two years after initiating ivacaftor and this is confirmed in our larger population.

We found changes in treatment patterns within the two pre-ivacaftor era cohorts tended to mirror each other, for example an increasing proportion of people on inhaled antibiotics between 2007-2012. The general increasing trend over time may reflect increasing age, changes in the quality of data capture for chronic medications within the Registry or changes in clinical practice over time.

We conducted a descriptive analysis. As such, we did not account for differences in the age/sex distribution of the different cohorts. However, the ivacaftor-treated group was on average older than the ineligible genotype group in the ivacaftor era, and the reduction in the proportions of individuals using other treatment over time is seen in spite of this. Furthermore, broadly similar patterns of treatment use were seen when we conditioned on sex, age and FEV_1_%. Our analyses were conducted by cohort rather than within-person. Further investigations could consider within-person treatment patterns. Furthermore, it will be of interest to estimate the impact of ivacaftor initiation on cessation of other treatments, with adjustment for confounding. One limitation of our study is that there have been a number of changes to the data capture of chronic medications within the UK CF Registry over the past decade, resulting in improved capture of use of chronic treatments in the registry. However, our comparison of genotype groups contemporaneously limits the impact of this on the interpretation of results. Further limitations include missing data, possible violations of our assumption that data were missing at random, and the possibility of inaccuracies in the data, such as misclassification of treatment status.

Understanding the association between ivacaftor use and ongoing prescription of other chronic treatments is important both from a treatment burden perspective and to appropriately inform health technology appraisals. National registries such as the UK CF Registry provide the opportunity to track these changes. The registry records whether a clinical care team includes a particular treatment for each patient in their list of current treatments (i.e. reflecting prescription), rather than whether the patient has actually been taking that treatment on a regular basis or indeed at all. Adherence levels are unknown and it is possible that the changes in treatment patterns over time may in fact be underestimated. Lower proportions of people prescribed chronic treatments in the ivacaftor group may reflect treatment discontinuation in response to no longer considering a therapy necessary, or recognition by the clinical team (and therefore removal from a current medication list) that the treatment is not being taken despite a clinical recommendation to do so. Treatment burden in CF is high ([8,13,14]) and ways to simplify this burden have been recognised as a priority area for clinical trials ([7,15]). The current rationale for discontinuing existing chronic treatments for patients on ivacaftor is not evidence based and the impact on clinical outcomes unknown.

The treatment differences we report within this observational data may be important in relation to longer-term clinical outcomes. It is of interest to investigate the impact of changes in use of other treatments on clinical outcomes in people taking ivacaftor, and to extend this when similar data become available from patients taking elexacaftor-tezacaftor-ivacaftor. Such investigations will complement the results that will be yielded from current or planned randomised controlled trials SIMPLIFY (ClinicalTrials.gov: NCT04378153) and CF STORM (EudraCT number 2020-005864-77), which aim to assess treatment simplification for mucoactive nebulised therapies in patients on elexacaftor-tezacaftor-ivacaftor. Understanding the association between change in treatment patterns and clinical outcomes is important to help interpret real-world effectiveness data on e.g. lung function outcomes of patients on CFTR modulators, particularly if there is any deviation from an anticipated degree of efficacy that may have been expected from the original phase III clinical trials. Multiple factors may contribute to this, but one possible explanation may be that treatment patterns for existing chronic therapies have not remained static. With the opportunity for significant potential benefits of highly effective modulators on long-term outcomes in people with CF, it is important that this is evaluated as a priority to help inform patients’ and clinical care team's decision making about long-term outcomes.

In conclusion, we have shown that there are clear differences in treatment patterns as documented on the CF Registry over a 5 year period for those treated with ivacaftor for G551D mutation, compared to a contemporary cohort not using ivacaftor. The methodology employed to compare groups provides an opportunity to understand the association of these changes with longer-term clinical outcomes.

## CRediT authorship contribution statement

**Emily Granger:** Methodology, Formal analysis, Visualization, Writing – original draft. **Gwyneth Davies:** Conceptualization, Methodology, Writing – original draft, Writing – review & editing, Supervision. **Ruth H. Keogh:** Conceptualization, Methodology, Writing – review & editing, Supervision, Funding acquisition.

## Appendix Supplementary materials

Image, application 1
