# Supplementary material for: Treatment patterns in people with cystic fibrosis: have they changed since the introduction of ivacaftor?
Source: J Cyst Fibros. 2022 Mar;21(2):316–22. doi: 10.1016/j.jcf.2021.08.014 (PMC9097695; doi:10.1016/j.jcf.2021.08.014)
Supplement: Supplementary file 1 [file mmc1.docx]

**SUPPLEMENTARY MATERIAL**

**TREATMENT PATTERNS IN PEOPLE WITH CYSTIC FIBROSIS: HAVE THEY CHANGED SINCE THE INTRODUCTION OF IVACAFTOR?**

Emily Granger^1^, Gwyneth Davies^2^, Ruth H. Keogh^1,3^

*^1^Department of Medical Statistics, Faculty of Epidemiology and Population Health, London School of Hygiene and Tropical Medicine, Keppel St, Bloomsbury, London WC1E 7HT*

*^2^ Population, Policy and Practice Research and Teaching Department, UCL Great Ormond Street Institute of Child Health, London, United Kingdom*

*^3^Centre for Statistical Methodology, London School of Hygiene and Tropical Medicine, Keppel St, Bloomsbury, London WC1E 7HT, United Kingdom*

This document includes a flowchart of patient selection for each cohort by year and tables presenting the proportions of each cohort taking different treatments by year along with the p-values from hypothesis tests for a difference in proportions at baseline and at the end of follow-up.

This supplement also contains results to compliment those presented in the main manuscript for subgroup analyses according to lung function, age and sex. Results are presented by cohort and then repeated separately for people with high, moderate and low lung function at baseline, children and adults, females and male. Additionally, this supplement includes a table of results for hypotheses tests of a difference in the trend of proportions of treatment use over time by genotype group, for the pre-ivacaftor era and ivacaftor era, separately.

| **List of table and figures** |
| --- |
| Supplementary Table 1: Proportions (and 95% confidence intervals) in each cohort taking different treatments by year. P-values from hypothesis test of a difference in proportions are also given for the baseline year and final follow-up year in each era. |
| Supplementary Table 2: Proportions (and 95% confidence intervals) in each cohort with high baseline lung function (FEV1%>80) taking different treatments by year. P-values from hypothesis test of a difference in proportions are also given for the baseline year and final follow-up year in each era. |
| Supplementary Table 3: Proportions (and 95% confidence intervals) in each cohort with moderate baseline lung function (<60FEV1%<80) taking different treatments by year. P-values from hypothesis test of a difference in proportions are also given for the baseline year and final follow-up year in each era. |
| Supplementary Table 4: Proportions (and 95% confidence intervals) in each cohort with low baseline lung function (FEV1%<60) taking different treatments by year. P-values from hypothesis test of a difference in proportions are also given for the baseline year and final follow-up year in each era. |
| Supplementary Table 5: Proportions (and 95% confidence intervals) in each cohort (children only) taking different treatments by year. P-values from hypothesis test of a difference in proportions are also given for the baseline year and final follow-up year in each era. |
| Supplementary Table 6: Proportions (and 95% confidence intervals) in each cohort (adults only) taking different treatments by year. P-values from hypothesis test of a difference in proportions are also given for the baseline year and final follow-up year in each era. |
| Supplementary Table 7: Proportions (and 95% confidence intervals) in each cohort (females only) taking different treatments by year. P-values from hypothesis test of a difference in proportions are also given for the baseline year and final follow-up year in each era. |
| Supplementary Table 8: Proportions (and 95% confidence intervals) in each cohort (males only) taking different treatments by year. P-values from hypothesis test of a difference in proportions are also given for the baseline year and final follow-up year in each era.  Supplementary Table 9: P-values associated with hypothesis tests for a difference in the trend of proportions of treatment use over time by genotype group. |
| Supplementary Figure 1: Flowchart of patient selection for each cohort  Supplementary Figure 2: The proportions and 95% confidence intervals in each cohort prescribed different treatments by year, stratified by FEV1% at baseline. |
| Supplementary Figure 3: The proportions and 95% confidence intervals in each cohort prescribed different treatments by year, stratified by sex. |
| Supplementary Figure 4: The proportions and 95% confidence intervals in each cohort prescribed different treatments by year, stratified by baseline age. |

*Supplementary Figure 1: Flow chart of patient selection for each cohort*

**
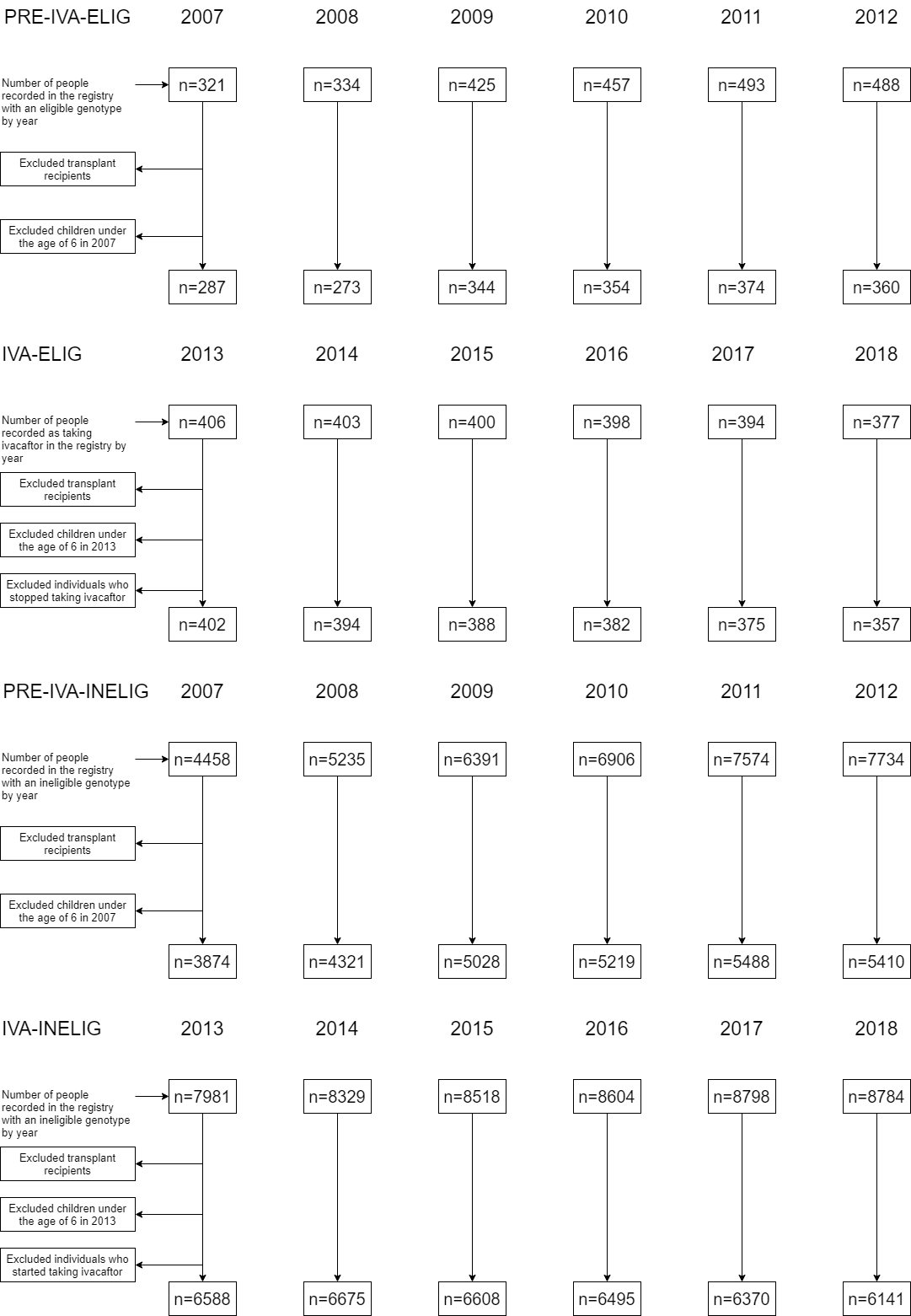
**

**Results according to FEV_1_% in the baseline year, sex and age**

In the pre-ivacaftor era treatment use is similar in both genotype groups across all three FEV_1_% subgroups. For inhaled antibiotics, dornase alfa and hypertonic saline solution, differences in treatment use between the two genotype groups at the end of the ivacaftor era are larger for people with high lung function (FEV1%>80), compared to those with moderate or low lung function. Inhaled antibiotic use was significantly lower in 2018 in the ivacaftor users group across all three FEV_1_% subgroups. Dornase alfa use and hypertonic saline use was significantly lower in 2018 in those with high or moderate FEV_1_%. Among people with low lung function (FEV_1_%$\leq$60), we only observed significant differences in the use of inhaled antibiotics and oral supplementary feeding between the ivacaftor treated group and the ineligible genotype group in 2018.

In the analysis by sex, we observed similar treatment patterns in the pre-ivacaftor era in both genotype groups in both males and females. In the ivacaftor era significant differences were observed between the two genotype groups in both males and females for inhaled antibiotics, dornase alfa, hypertonic saline solution, and oral supplementary feeding. For oral supplementary feeding, in females the proportion of users remained approximately constant during the ivacaftor era in the ivacaftor treated group and in the ineligible genotype group, though the proportion of users was lower in the baseline year (2013) in the ivacaftor treated group. For chronic oral antibiotics and flucloxacillin we found a significant difference between the genotype groups in 2018 for males only, while for azithromycin and gastrostomy supplementary feeding we saw a significant difference between the genotype groups in 2018 for females only.

For inhaled antibiotics, dornase alfa, and hypertonic saline solution we observed larger differences in treatment use between genotype groups in the ivacaftor-era among children, compared to adults. There were significant differences in the use of inhaled antibiotics, dornase alfa, hypertonic saline solution, and oral supplementary in both children and adults. In adults there was also evidence of a difference in the proportions using chronic oral antibiotics and supplementary feeding by gastrostomy in the ivacaftor treated group compared to the ineligible genotype group in 2018, and some evidence of a difference in flucloxacillin use and supplementary feeding by gastrostomy. However, use of these treatments was also lower in the ivacaftor treated group in the baseline year (2013). In children, in the ivacaftor era, azithromycin use was significantly higher in the ineligible genotype group in 2018 compared to the ivacaftor treated group, whereas there was no difference in adults.

| Supplementary Table 1: Proportions (and 95% confidence intervals) in each cohort taking different treatments by year. P-values from hypothesis test of a difference in proportions are also given for the baseline year and final follow-up year in each era. | | | | | | | | | | | | |
| --- | --- | --- | --- | --- | --- | --- | --- | --- | --- | --- | --- | --- |
|  | Pre-ivacaftor era | | | | | | Post-ivacaftor era | | | | | |
|  | 2007 | 2008 | 2009 | 2010 | 2011 | 2012 | 2013 | 2014 | 2015 | 2016 | 2017 | 2018 |
| Inhaled Antibiotics | | | | | | | | | | | | |
| Eligible | 0.45 | 0.53 | 0.53 | 0.57 | 0.61 | 0.65 | 0.65 | 0.62 | 0.58 | 0.49 | 0.43 | 0.40 |
|  | (0.4,0.51) | (0.47,0.59) | (0.48,0.58) | (0.52,0.62) | (0.56,0.66) | (0.6,0.7) | (0.6,0.69) | (0.57,0.67) | (0.53,0.63) | (0.44,0.54) | (0.38,0.48) | (0.35,0.45) |
| Ineligible | 0.52 | 0.59 | 0.57 | 0.61 | 0.63 | 0.66 | 0.65 | 0.67 | 0.65 | 0.56 | 0.56 | 0.56 |
|  | (0.5,0.54) | (0.58,0.61) | (0.56,0.58) | (0.6,0.63) | (0.62,0.65) | (0.64,0.67) | (0.64,0.66) | (0.66,0.68) | (0.63,0.66) | (0.55,0.57) | (0.55,0.58) | (0.55,0.58) |
| p-values | 0.035 |  |  |  |  | 0.745 | 0.949 |  |  |  |  | <0.001 |
| Dornase alfa | | | | | | | | | | | | |
| Eligible | 0.30 | 0.40 | 0.42 | 0.52 | 0.50 | 0.53 | 0.60 | 0.59 | 0.60 | 0.57 | 0.56 | 0.57 |
|  | (0.24,0.35) | (0.34,0.46) | (0.37,0.47) | (0.47,0.57) | (0.45,0.55) | (0.48,0.58) | (0.55,0.64) | (0.54,0.64) | (0.55,0.65) | (0.52,0.62) | (0.51,0.61) | (0.52,0.63) |
| Ineligible | 0.35 | 0.45 | 0.47 | 0.53 | 0.56 | 0.60 | 0.62 | 0.67 | 0.70 | 0.70 | 0.74 | 0.76 |
|  | (0.33,0.36) | (0.43,0.46) | (0.46,0.48) | (0.52,0.54) | (0.55,0.57) | (0.59,0.61) | (0.6,0.63) | (0.65,0.68) | (0.69,0.71) | (0.69,0.71) | (0.73,0.75) | (0.75,0.77) |
| p-values | 0.0924 |  |  |  |  | 0.015 | 0.466 |  |  |  |  | <0.001 |
| Hypertonic saline solution | | | | | | | | | | | | |
| Eligible | 0.03 | 0.05 | 0.06 | 0.12 | 0.17 | 0.25 | 0.29 | 0.30 | 0.28 | 0.27 | 0.26 | 0.24 |
|  | (0.01,0.05) | (0.02,0.07) | (0.03,0.08) | (0.09,0.16) | (0.13,0.2) | (0.2,0.29) | (0.25,0.34) | (0.25,0.34) | (0.23,0.32) | (0.23,0.32) | (0.22,0.31) | (0.19,0.28) |
| Ineligible | 0.05 | 0.07 | 0.09 | 0.15 | 0.20 | 0.25 | 0.29 | 0.34 | 0.36 | 0.35 | 0.37 | 0.39 |
|  | (0.04,0.06) | (0.06,0.08) | (0.08,0.1) | (0.14,0.16) | (0.19,0.22) | (0.24,0.27) | (0.28,0.3) | (0.33,0.35) | (0.35,0.37) | (0.34,0.36) | (0.36,0.38) | (0.38,0.4) |
| p-values | 0.219 |  |  |  |  | 0.794 | 0.979 |  |  |  |  | <0.001 |
| Chronic oral antibiotics | | | | | | | | | | | | |
| Eligible | 0.40 | 0.46 | 0.44 | 0.44 | 0.44 | 0.43 | 0.49 | 0.50 | 0.48 | 0.40 | 0.38 | 0.37 |
|  | (0.34,0.46) | (0.4,0.52) | (0.39,0.49) | (0.39,0.49) | (0.39,0.49) | (0.38,0.48) | (0.44,0.53) | (0.45,0.55) | (0.43,0.53) | (0.35,0.44) | (0.33,0.43) | (0.32,0.42) |
| Ineligible | 0.41 | 0.42 | 0.43 | 0.44 | 0.43 | 0.44 | 0.50 | 0.53 | 0.52 | 0.48 | 0.46 | 0.45 |
|  | (0.4,0.43) | (0.4,0.43) | (0.42,0.44) | (0.42,0.45) | (0.41,0.44) | (0.43,0.46) | (0.49,0.51) | (0.52,0.54) | (0.51,0.53) | (0.47,0.5) | (0.45,0.47) | (0.44,0.46) |
| p-values | 0.729 |  |  |  |  | 0.750 | 0.676 |  |  |  |  | 0.004 |
| Azithromycin | | | | | | | | | | | | |
| Eligible | 0.26 | 0.37 | 0.43 | 0.51 | 0.54 | 0.55 | 0.50 | 0.46 | 0.48 | 0.44 | 0.46 | 0.46 |
|  | (0.21,0.32) | (0.32,0.43) | (0.38,0.49) | (0.46,0.56) | (0.49,0.59) | (0.5,0.6) | (0.45,0.55) | (0.41,0.51) | (0.43,0.53) | (0.39,0.49) | (0.41,0.51) | (0.41,0.51) |
| Ineligible | 0.28 | 0.41 | 0.43 | 0.51 | 0.54 | 0.51 | 0.48 | 0.47 | 0.48 | 0.49 | 0.53 | 0.54 |
|  | (0.27,0.29) | (0.4,0.43) | (0.42,0.45) | (0.5,0.53) | (0.53,0.56) | (0.5,0.53) | (0.47,0.49) | (0.46,0.49) | (0.47,0.49) | (0.47,0.5) | (0.51,0.54) | (0.52,0.55) |
| p-values | 0.612 |  |  |  |  | 0.203 | 0.403 |  |  |  |  | 0.005 |
| Flucloxacillin | | | | | | | | | | | | |
| Eligible | 0.29 | 0.32 | 0.30 | 0.31 | 0.29 | 0.26 | 0.33 | 0.35 | 0.33 | 0.27 | 0.26 | 0.25 |
|  | (0.23,0.34) | (0.27,0.38) | (0.25,0.35) | (0.27,0.36) | (0.24,0.33) | (0.22,0.31) | (0.29,0.38) | (0.3,0.39) | (0.29,0.38) | (0.22,0.31) | (0.22,0.31) | (0.2,0.29) |
| Ineligible | 0.28 | 0.30 | 0.30 | 0.31 | 0.30 | 0.30 | 0.34 | 0.34 | 0.33 | 0.30 | 0.29 | 0.27 |
|  | (0.27,0.29) | (0.29,0.31) | (0.29,0.32) | (0.3,0.33) | (0.29,0.32) | (0.28,0.31) | (0.33,0.35) | (0.33,0.35) | (0.32,0.35) | (0.28,0.31) | (0.28,0.3) | (0.26,0.29) |
| p-values | 0.898 |  |  |  |  | 0.183 | 0.855 |  |  |  |  | 0.277 |
| Supplementary feeding (oral) | | | | | | | | | | | | |
| Eligible | 0.16 | 0.20 | 0.26 | 0.24 | 0.25 | 0.27 | 0.23 | 0.20 | 0.23 | 0.18 | 0.19 | 0.22 |
|  | (0.12,0.21) | (0.15,0.25) | (0.21,0.3) | (0.2,0.29) | (0.21,0.3) | (0.22,0.31) | (0.19,0.27) | (0.16,0.24) | (0.19,0.27) | (0.14,0.21) | (0.15,0.23) | (0.17,0.26) |
| Ineligible | 0.18 | 0.25 | 0.26 | 0.26 | 0.27 | 0.28 | 0.28 | 0.29 | 0.30 | 0.30 | 0.29 | 0.31 |
|  | (0.17,0.19) | (0.24,0.27) | (0.25,0.27) | (0.24,0.27) | (0.26,0.28) | (0.27,0.29) | (0.27,0.29) | (0.28,0.31) | (0.29,0.32) | (0.29,0.31) | (0.28,0.3) | (0.29,0.32) |
| p-values | 0.586 |  |  |  |  | 0.659 | 0.022 |  |  |  |  | <0.001 |
| Supplementary feeding (gastrostomy) | | | | | | | | | | | | |
| Eligible | 0.02 | 0.04 | 0.06 | 0.06 | 0.06 | 0.07 | 0.07 | 0.05 | 0.04 | 0.04 | 0.03 | 0.03 |
|  | (0.01,0.04) | (0.01,0.06) | (0.03,0.08) | (0.03,0.08) | (0.04,0.09) | (0.05,0.1) | (0.04,0.09) | (0.03,0.07) | (0.02,0.06) | (0.02,0.06) | (0.02,0.05) | (0.01,0.05) |
| Ineligible | 0.04 | 0.07 | 0.07 | 0.07 | 0.07 | 0.07 | 0.07 | 0.07 | 0.07 | 0.06 | 0.06 | 0.07 |
|  | (0.03,0.05) | (0.06,0.07) | (0.06,0.08) | (0.07,0.08) | (0.07,0.08) | (0.07,0.08) | (0.06,0.07) | (0.06,0.08) | (0.06,0.07) | (0.06,0.07) | (0.06,0.07) | (0.06,0.07) |
| p-values | 0.231 |  |  |  |  | 1 | 1 |  |  |  |  | 0.020 |

| Supplementary Table 2: Proportions (and 95% confidence intervals) in each cohort with high baseline lung function (FEV1%>80) taking different treatments by year. P-values from hypothesis test of a difference in proportions are also given for the baseline year and final follow-up year in each era. | | | | | | | | | | | | |
| --- | --- | --- | --- | --- | --- | --- | --- | --- | --- | --- | --- | --- |
|  | Pre-ivacaftor era | | | | | | Post-ivacaftor era | | | | | |
|  | 2007 | 2008 | 2009 | 2010 | 2011 | 2012 | 2013 | 2014 | 2015 | 2016 | 2017 | 2018 |
| Inhaled Antibiotics | | | | | | | | | | | | |
| Eligible | 0.36 | 0.49 | 0.47 | 0.50 | 0.53 | 0.60 | 0.54 | 0.50 | 0.43 | 0.37 | 0.35 | 0.32 |
|  | (0.27,0.46) | (0.38,0.6) | (0.36,0.57) | (0.4,0.6) | (0.42,0.63) | (0.49,0.7) | (0.47,0.61) | (0.43,0.58) | (0.36,0.5) | (0.3,0.45) | (0.28,0.42) | (0.25,0.39) |
| Ineligible | 0.47 | 0.52 | 0.51 | 0.58 | 0.60 | 0.62 | 0.50 | 0.54 | 0.55 | 0.47 | 0.49 | 0.49 |
|  | (0.44,0.49) | (0.49,0.55) | (0.48,0.54) | (0.55,0.61) | (0.57,0.63) | (0.59,0.65) | (0.48,0.52) | (0.52,0.56) | (0.53,0.57) | (0.45,0.49) | (0.47,0.51) | (0.47,0.51) |
| p-values | 0.061 |  |  |  |  | 0.738 | 0.347 |  |  |  |  | <0.001 |
| Dornase alfa | | | | | | | | | | | | |
| Eligible | 0.22 | 0.30 | 0.28 | 0.39 | 0.43 | 0.49 | 0.48 | 0.47 | 0.45 | 0.43 | 0.46 | 0.48 |
|  | (0.13,0.3) | (0.21,0.4) | (0.19,0.38) | (0.29,0.49) | (0.33,0.53) | (0.39,0.6) | (0.41,0.56) | (0.4,0.55) | (0.38,0.53) | (0.35,0.5) | (0.38,0.53) | (0.4,0.55) |
| Ineligible | 0.21 | 0.27 | 0.32 | 0.39 | 0.45 | 0.51 | 0.47 | 0.56 | 0.62 | 0.65 | 0.70 | 0.73 |
|  | (0.19,0.24) | (0.25,0.3) | (0.29,0.34) | (0.36,0.42) | (0.42,0.48) | (0.48,0.54) | (0.45,0.49) | (0.54,0.58) | (0.6,0.64) | (0.63,0.67) | (0.68,0.72) | (0.71,0.75) |
| p-values | 1 |  |  |  |  | 0.895 | 0.833 |  |  |  |  | <0.001 |
| Hypertonic saline solution | | | | | | | | | | | | |
| Eligible | 0.01 | 0.05 | 0.02 | 0.09 | 0.13 | 0.15 | 0.18 | 0.19 | 0.17 | 0.17 | 0.16 | 0.14 |
|  | (0,0.03) | (0,0.1) | (0,0.05) | (0.03,0.15) | (0.06,0.2) | (0.07,0.22) | (0.13,0.24) | (0.13,0.25) | (0.11,0.22) | (0.11,0.23) | (0.1,0.22) | (0.09,0.19) |
| Ineligible | 0.02 | 0.04 | 0.05 | 0.11 | 0.15 | 0.21 | 0.21 | 0.25 | 0.29 | 0.31 | 0.33 | 0.37 |
|  | (0.01,0.03) | (0.03,0.05) | (0.04,0.07) | (0.09,0.13) | (0.13,0.18) | (0.19,0.24) | (0.19,0.23) | (0.23,0.27) | (0.28,0.31) | (0.29,0.33) | (0.31,0.35) | (0.35,0.39) |
| p-values | 0.697 |  |  |  |  | 0.202 | 0.487 |  |  |  |  | <0.001 |
| Chronic oral antibiotics | | | | | | | | | | | | |
| Eligible | 0.51 | 0.62 | 0.55 | 0.50 | 0.53 | 0.51 | 0.53 | 0.54 | 0.53 | 0.43 | 0.44 | 0.38 |
|  | (0.41,0.6) | (0.52,0.73) | (0.44,0.65) | (0.4,0.6) | (0.42,0.63) | (0.4,0.61) | (0.46,0.6) | (0.46,0.61) | (0.45,0.6) | (0.36,0.51) | (0.36,0.51) | (0.31,0.46) |
| Ineligible | 0.45 | 0.45 | 0.46 | 0.46 | 0.46 | 0.46 | 0.55 | 0.58 | 0.57 | 0.52 | 0.48 | 0.48 |
|  | (0.42,0.48) | (0.42,0.48) | (0.42,0.49) | (0.43,0.49) | (0.42,0.49) | (0.43,0.5) | (0.53,0.57) | (0.56,0.6) | (0.55,0.59) | (0.5,0.54) | (0.46,0.5) | (0.46,0.5) |
| p-values | 0.313 |  |  |  |  | 0.531 | 0.652 |  |  |  |  | 0.025 |
| Azithromycin | | | | | | | | | | | | |
| Eligible | 0.14 | 0.27 | 0.33 | 0.30 | 0.42 | 0.40 | 0.39 | 0.34 | 0.35 | 0.31 | 0.36 | 0.35 |
|  | (0.07,0.21) | (0.17,0.36) | (0.23,0.43) | (0.21,0.39) | (0.32,0.52) | (0.3,0.51) | (0.32,0.46) | (0.27,0.41) | (0.28,0.42) | (0.24,0.38) | (0.29,0.43) | (0.28,0.43) |
| Ineligible | 0.15 | 0.23 | 0.29 | 0.38 | 0.43 | 0.41 | 0.30 | 0.31 | 0.32 | 0.33 | 0.38 | 0.40 |
|  | (0.13,0.17) | (0.2,0.26) | (0.27,0.32) | (0.35,0.41) | (0.4,0.46) | (0.38,0.44) | (0.29,0.32) | (0.29,0.33) | (0.3,0.34) | (0.31,0.35) | (0.36,0.4) | (0.38,0.42) |
| p-values | 0.942 |  |  |  |  | 0.973 | 0.018 |  |  |  |  | 0.257 |
| Flucloxacillin | | | | | | | | | | | | |
| Eligible | 0.36 | 0.44 | 0.38 | 0.34 | 0.35 | 0.36 | 0.41 | 0.39 | 0.38 | 0.31 | 0.31 | 0.26 |
|  | (0.27,0.46) | (0.33,0.55) | (0.27,0.48) | (0.25,0.44) | (0.25,0.45) | (0.26,0.46) | (0.34,0.48) | (0.32,0.46) | (0.31,0.45) | (0.24,0.38) | (0.24,0.38) | (0.2,0.33) |
| Ineligible | 0.30 | 0.31 | 0.31 | 0.31 | 0.31 | 0.30 | 0.38 | 0.39 | 0.38 | 0.33 | 0.32 | 0.31 |
|  | (0.27,0.33) | (0.28,0.34) | (0.28,0.34) | (0.29,0.34) | (0.28,0.33) | (0.27,0.33) | (0.36,0.4) | (0.37,0.41) | (0.36,0.4) | (0.31,0.35) | (0.3,0.34) | (0.29,0.33) |
| p-values | 0.241 |  |  |  |  | 0.312 | 0.564 |  |  |  |  | 0.292 |
| Supplementary feeding (oral) | | | | | | | | | | | | |
| Eligible | 0.19 | 0.18 | 0.22 | 0.18 | 0.22 | 0.24 | 0.16 | 0.12 | 0.18 | 0.14 | 0.11 | 0.16 |
|  | (0.11,0.26) | (0.1,0.27) | (0.13,0.3) | (0.1,0.26) | (0.13,0.3) | (0.15,0.33) | (0.11,0.22) | (0.08,0.17) | (0.12,0.23) | (0.09,0.19) | (0.06,0.16) | (0.11,0.22) |
| Ineligible | 0.15 | 0.17 | 0.17 | 0.19 | 0.19 | 0.20 | 0.19 | 0.20 | 0.21 | 0.22 | 0.21 | 0.24 |
|  | (0.13,0.17) | (0.15,0.2) | (0.15,0.19) | (0.16,0.21) | (0.17,0.22) | (0.18,0.23) | (0.17,0.2) | (0.18,0.21) | (0.19,0.23) | (0.2,0.24) | (0.2,0.23) | (0.22,0.26) |
| p-values | 0.478 |  |  |  |  | 0.473 | 0.469 |  |  |  |  | 0.022 |
| Supplementary feeding (gastrostomy) | | | | | | | | | | | | |
| Eligible | 0.00 | 0.00 | 0.02 | 0.02 | 0.03 | 0.03 | 0.02 | 0.01 | 0.01 | 0.01 | 0.01 | 0.01 |
|  | (0,0) | (0,0) | (0,0.05) | (0,0.05) | (0,0.07) | (0,0.07) | (0,0.03) | (0,0.03) | (0,0.03) | (0,0.03) | (0,0.02) | (0,0.02) |
| Ineligible | 0.03 | 0.03 | 0.03 | 0.03 | 0.03 | 0.04 | 0.02 | 0.02 | 0.02 | 0.02 | 0.02 | 0.03 |
|  | (0.02,0.04) | (0.02,0.04) | (0.02,0.04) | (0.02,0.05) | (0.02,0.05) | (0.02,0.05) | (0.01,0.02) | (0.01,0.02) | (0.01,0.02) | (0.02,0.03) | (0.02,0.03) | (0.02,0.03) |
| p-values | 0.169 |  |  |  |  | 1 | 1 |  |  |  |  | 0.162 |

| Supplementary Table 3: Proportions (and 95% confidence intervals) in each cohort with moderate baseline lung function (<60FEV1%<80) taking different treatments by year. P-values from hypothesis test of a difference in proportions are also given for the baseline year and final follow-up year in each era. | | | | | | | | | | | | |
| --- | --- | --- | --- | --- | --- | --- | --- | --- | --- | --- | --- | --- |
|  | Pre-ivacaftor era | | | | | | Post-ivacaftor era | | | | | |
|  | 2007 | 2008 | 2009 | 2010 | 2011 | 2012 | 2013 | 2014 | 2015 | 2016 | 2017 | 2018 |
| Inhaled Antibiotics | | | | | | | | | | | | |
| Eligible | 0.55 | 0.58 | 0.55 | 0.57 | 0.68 | 0.69 | 0.73 | 0.67 | 0.69 | 0.52 | 0.44 | 0.43 |
|  | (0.43,0.67) | (0.45,0.71) | (0.43,0.67) | (0.45,0.69) | (0.56,0.79) | (0.57,0.81) | (0.64,0.82) | (0.58,0.77) | (0.59,0.78) | (0.42,0.63) | (0.34,0.55) | (0.33,0.54) |
| Ineligible | 0.56 | 0.63 | 0.62 | 0.69 | 0.71 | 0.75 | 0.69 | 0.70 | 0.68 | 0.58 | 0.57 | 0.59 |
|  | (0.53,0.6) | (0.6,0.67) | (0.59,0.65) | (0.66,0.73) | (0.68,0.74) | (0.72,0.78) | (0.67,0.71) | (0.68,0.72) | (0.66,0.71) | (0.56,0.6) | (0.55,0.6) | (0.56,0.61) |
| p-values | 0.958 |  |  |  |  | 0.393 | 0.432 |  |  |  |  | 0.007 |
| Dornase alfa | | | | | | | | | | | | |
| Eligible | 0.31 | 0.35 | 0.37 | 0.46 | 0.48 | 0.52 | 0.64 | 0.66 | 0.69 | 0.63 | 0.58 | 0.63 |
|  | (0.2,0.42) | (0.22,0.47) | (0.25,0.49) | (0.34,0.58) | (0.36,0.61) | (0.39,0.65) | (0.55,0.74) | (0.56,0.76) | (0.59,0.78) | (0.53,0.73) | (0.48,0.69) | (0.52,0.73) |
| Ineligible | 0.35 | 0.46 | 0.48 | 0.56 | 0.60 | 0.65 | 0.66 | 0.71 | 0.75 | 0.75 | 0.78 | 0.80 |
|  | (0.32,0.38) | (0.42,0.49) | (0.45,0.51) | (0.53,0.59) | (0.57,0.63) | (0.62,0.68) | (0.64,0.68) | (0.68,0.73) | (0.73,0.77) | (0.73,0.77) | (0.76,0.8) | (0.78,0.81) |
| p-values | 0.603 |  |  |  |  | 0.053 | 0.875 |  |  |  |  | <0.001 |
| Hypertonic saline solution | | | | | | | | | | | | |
| Eligible | 0.00 | 0.00 | 0.05 | 0.08 | 0.13 | 0.22 | 0.31 | 0.30 | 0.29 | 0.28 | 0.30 | 0.28 |
|  | (0,0) | (0,0) | (0,0.1) | (0.01,0.15) | (0.05,0.21) | (0.12,0.33) | (0.22,0.41) | (0.21,0.4) | (0.2,0.39) | (0.18,0.37) | (0.21,0.4) | (0.18,0.37) |
| Ineligible | 0.04 | 0.07 | 0.10 | 0.16 | 0.22 | 0.28 | 0.31 | 0.35 | 0.39 | 0.37 | 0.39 | 0.40 |
|  | (0.03,0.05) | (0.05,0.09) | (0.08,0.12) | (0.14,0.19) | (0.19,0.25) | (0.25,0.31) | (0.29,0.33) | (0.33,0.38) | (0.37,0.41) | (0.35,0.39) | (0.37,0.41) | (0.38,0.43) |
| p-values | 0.120 |  |  |  |  | 0.437 | 1 |  |  |  |  | 0.032 |
| Chronic oral antibiotics | | | | | | | | | | | | |
| Eligible | 0.43 | 0.45 | 0.45 | 0.43 | 0.40 | 0.38 | 0.50 | 0.51 | 0.47 | 0.35 | 0.36 | 0.39 |
|  | (0.31,0.55) | (0.32,0.59) | (0.33,0.57) | (0.31,0.55) | (0.28,0.53) | (0.25,0.5) | (0.4,0.6) | (0.4,0.61) | (0.37,0.58) | (0.25,0.45) | (0.26,0.46) | (0.28,0.49) |
| Ineligible | 0.43 | 0.42 | 0.45 | 0.44 | 0.44 | 0.45 | 0.51 | 0.56 | 0.54 | 0.51 | 0.47 | 0.46 |
|  | (0.4,0.46) | (0.38,0.45) | (0.42,0.48) | (0.41,0.47) | (0.41,0.47) | (0.42,0.48) | (0.49,0.54) | (0.53,0.58) | (0.52,0.57) | (0.49,0.54) | (0.45,0.49) | (0.43,0.48) |
| p-values | 1 |  |  |  |  | 0.256 | 0.894 |  |  |  |  | 0.251 |
| Azithromycin | | | | | | | | | | | | |
| Eligible | 0.34 | 0.45 | 0.46 | 0.63 | 0.66 | 0.66 | 0.52 | 0.52 | 0.54 | 0.44 | 0.47 | 0.51 |
|  | (0.23,0.46) | (0.32,0.59) | (0.34,0.58) | (0.52,0.75) | (0.54,0.78) | (0.53,0.78) | (0.42,0.63) | (0.41,0.62) | (0.44,0.64) | (0.34,0.55) | (0.36,0.57) | (0.4,0.61) |
| Ineligible | 0.27 | 0.44 | 0.48 | 0.56 | 0.59 | 0.57 | 0.49 | 0.50 | 0.52 | 0.53 | 0.57 | 0.59 |
|  | (0.24,0.3) | (0.4,0.47) | (0.44,0.51) | (0.53,0.59) | (0.56,0.62) | (0.53,0.6) | (0.47,0.52) | (0.48,0.53) | (0.5,0.54) | (0.51,0.56) | (0.55,0.59) | (0.56,0.61) |
| p-values | 0.242 |  |  |  |  | 0.176 | 0.688 |  |  |  |  | 0.234 |
| Flucloxacillin | | | | | | | | | | | | |
| Eligible | 0.27 | 0.25 | 0.26 | 0.27 | 0.24 | 0.16 | 0.33 | 0.34 | 0.33 | 0.27 | 0.27 | 0.28 |
|  | (0.16,0.37) | (0.14,0.37) | (0.15,0.37) | (0.16,0.38) | (0.14,0.35) | (0.06,0.25) | (0.24,0.43) | (0.24,0.44) | (0.23,0.42) | (0.17,0.36) | (0.17,0.36) | (0.18,0.37) |
| Ineligible | 0.29 | 0.29 | 0.31 | 0.30 | 0.29 | 0.28 | 0.35 | 0.35 | 0.35 | 0.31 | 0.29 | 0.27 |
|  | (0.26,0.32) | (0.26,0.32) | (0.28,0.34) | (0.27,0.34) | (0.26,0.32) | (0.25,0.31) | (0.33,0.37) | (0.33,0.37) | (0.32,0.37) | (0.29,0.33) | (0.27,0.31) | (0.24,0.29) |
| p-values | 0.817 |  |  |  |  | 0.050 | 0.851 |  |  |  |  | 0.925 |
| Supplementary feeding (oral) | | | | | | | | | | | | |
| Eligible | 0.16 | 0.18 | 0.18 | 0.21 | 0.19 | 0.28 | 0.22 | 0.15 | 0.24 | 0.21 | 0.22 | 0.28 |
|  | (0.08,0.25) | (0.08,0.28) | (0.09,0.28) | (0.11,0.31) | (0.1,0.29) | (0.16,0.39) | (0.14,0.31) | (0.07,0.22) | (0.15,0.32) | (0.12,0.3) | (0.13,0.31) | (0.18,0.37) |
| Ineligible | 0.20 | 0.22 | 0.23 | 0.25 | 0.25 | 0.27 | 0.27 | 0.29 | 0.31 | 0.31 | 0.30 | 0.32 |
|  | (0.17,0.22) | (0.19,0.25) | (0.2,0.26) | (0.22,0.28) | (0.22,0.28) | (0.24,0.3) | (0.25,0.29) | (0.27,0.31) | (0.29,0.33) | (0.29,0.33) | (0.28,0.33) | (0.3,0.34) |
| p-values | 0.598 |  |  |  |  | 1 | 0.429 |  |  |  |  | 0.468 |
| Supplementary feeding (gastrostomy) | | | | | | | | | | | | |
| Eligible | 0.03 | 0.04 | 0.03 | 0.03 | 0.06 | 0.07 | 0.08 | 0.04 | 0.02 | 0.01 | 0.03 | 0.02 |
|  | (0,0.07) | (0,0.09) | (0,0.07) | (0,0.08) | (0,0.13) | (0,0.13) | (0.02,0.13) | (0,0.09) | (0,0.05) | (0,0.03) | (0,0.07) | (0,0.06) |
| Ineligible | 0.03 | 0.04 | 0.05 | 0.06 | 0.06 | 0.06 | 0.05 | 0.05 | 0.06 | 0.06 | 0.06 | 0.06 |
|  | (0.02,0.05) | (0.02,0.05) | (0.03,0.06) | (0.05,0.08) | (0.05,0.08) | (0.04,0.07) | (0.04,0.06) | (0.04,0.06) | (0.05,0.07) | (0.05,0.07) | (0.05,0.07) | (0.05,0.08) |
| p-values | 1 |  |  |  |  | 0.945 | 0.355 |  |  |  |  | 0.209 |

| Supplementary Table 4: Proportions (and 95% confidence intervals) in each cohort with low baseline lung function (FEV1%<60) taking different treatments by year. P-values from hypothesis test of a difference in proportions are also given for the baseline year and final follow-up year in each era. | | | | | | | | | | | | |
| --- | --- | --- | --- | --- | --- | --- | --- | --- | --- | --- | --- | --- |
|  | Pre-ivacaftor era | | | | | | Post-ivacaftor era | | | | | |
|  | 2007 | 2008 | 2009 | 2010 | 2011 | 2012 | 2013 | 2014 | 2015 | 2016 | 2017 | 2018 |
| Inhaled Antibiotics | | | | | | | | | | | | |
| Eligible | 0.58 | 0.63 | 0.70 | 0.73 | 0.78 | 0.80 | 0.76 | 0.76 | 0.74 | 0.66 | 0.57 | 0.52 |
|  | (0.47,0.7) | (0.5,0.76) | (0.58,0.82) | (0.62,0.85) | (0.67,0.89) | (0.69,0.91) | (0.69,0.84) | (0.68,0.84) | (0.66,0.82) | (0.57,0.75) | (0.47,0.67) | (0.41,0.62) |
| Ineligible | 0.61 | 0.66 | 0.69 | 0.73 | 0.77 | 0.81 | 0.80 | 0.82 | 0.78 | 0.71 | 0.70 | 0.70 |
|  | (0.58,0.64) | (0.63,0.7) | (0.66,0.72) | (0.69,0.76) | (0.74,0.8) | (0.78,0.84) | (0.78,0.82) | (0.8,0.83) | (0.77,0.8) | (0.69,0.74) | (0.68,0.72) | (0.67,0.72) |
| p-values | 0.749 |  |  |  |  | 1 | 0.398 |  |  |  |  | 0.001 |
| Dornase alfa | | | | | | | | | | | | |
| Eligible | 0.46 | 0.57 | 0.60 | 0.71 | 0.65 | 0.78 | 0.72 | 0.70 | 0.76 | 0.73 | 0.71 | 0.71 |
|  | (0.34,0.57) | (0.44,0.71) | (0.48,0.72) | (0.6,0.83) | (0.53,0.78) | (0.67,0.9) | (0.64,0.8) | (0.61,0.79) | (0.68,0.84) | (0.65,0.82) | (0.62,0.8) | (0.61,0.8) |
| Ineligible | 0.52 | 0.57 | 0.62 | 0.66 | 0.68 | 0.72 | 0.76 | 0.80 | 0.80 | 0.77 | 0.79 | 0.80 |
|  | (0.49,0.55) | (0.54,0.61) | (0.59,0.66) | (0.62,0.69) | (0.65,0.71) | (0.68,0.75) | (0.74,0.78) | (0.78,0.81) | (0.78,0.82) | (0.76,0.79) | (0.77,0.81) | (0.78,0.82) |
| p-values | 0.389 |  |  |  |  | 0.376 | 0.383 |  |  |  |  | 0.063 |
| Hypertonic saline solution | | | | | | | | | | | | |
| Eligible | 0.07 | 0.11 | 0.12 | 0.12 | 0.22 | 0.27 | 0.46 | 0.45 | 0.41 | 0.39 | 0.39 | 0.36 |
|  | (0.01,0.13) | (0.03,0.19) | (0.04,0.2) | (0.04,0.21) | (0.11,0.33) | (0.15,0.4) | (0.37,0.55) | (0.35,0.54) | (0.32,0.51) | (0.3,0.49) | (0.29,0.49) | (0.26,0.47) |
| Ineligible | 0.08 | 0.12 | 0.16 | 0.23 | 0.27 | 0.32 | 0.38 | 0.43 | 0.43 | 0.40 | 0.42 | 0.42 |
|  | (0.07,0.1) | (0.1,0.14) | (0.13,0.18) | (0.2,0.26) | (0.24,0.3) | (0.28,0.35) | (0.36,0.4) | (0.41,0.45) | (0.41,0.45) | (0.37,0.42) | (0.4,0.44) | (0.39,0.44) |
| p-values | 0.831 |  |  |  |  | 0.619 | 0.102 |  |  |  |  | 0.379 |
| Chronic oral antibiotics | | | | | | | | | | | | |
| Eligible | 0.35 | 0.37 | 0.40 | 0.45 | 0.45 | 0.49 | 0.41 | 0.43 | 0.40 | 0.35 | 0.30 | 0.31 |
|  | (0.24,0.46) | (0.24,0.5) | (0.28,0.52) | (0.32,0.58) | (0.32,0.59) | (0.35,0.63) | (0.32,0.5) | (0.33,0.52) | (0.31,0.5) | (0.25,0.44) | (0.21,0.39) | (0.21,0.4) |
| Ineligible | 0.43 | 0.39 | 0.42 | 0.39 | 0.38 | 0.42 | 0.43 | 0.46 | 0.45 | 0.43 | 0.42 | 0.40 |
|  | (0.39,0.46) | (0.36,0.43) | (0.38,0.45) | (0.35,0.42) | (0.35,0.42) | (0.39,0.46) | (0.41,0.45) | (0.44,0.49) | (0.43,0.48) | (0.41,0.46) | (0.39,0.44) | (0.38,0.43) |
| p-values | 0.238 |  |  |  |  | 0.427 | 0.719 |  |  |  |  | 0.101 |
| Azithromycin | | | | | | | | | | | | |
| Eligible | 0.38 | 0.46 | 0.60 | 0.64 | 0.65 | 0.63 | 0.65 | 0.63 | 0.65 | 0.63 | 0.66 | 0.65 |
|  | (0.26,0.49) | (0.33,0.6) | (0.48,0.72) | (0.52,0.77) | (0.53,0.78) | (0.49,0.76) | (0.57,0.74) | (0.54,0.72) | (0.56,0.75) | (0.54,0.73) | (0.57,0.75) | (0.55,0.75) |
| Ineligible | 0.48 | 0.61 | 0.65 | 0.71 | 0.73 | 0.71 | 0.67 | 0.66 | 0.66 | 0.67 | 0.71 | 0.71 |
|  | (0.44,0.51) | (0.58,0.64) | (0.62,0.68) | (0.68,0.74) | (0.7,0.76) | (0.67,0.74) | (0.65,0.69) | (0.64,0.68) | (0.64,0.68) | (0.65,0.7) | (0.69,0.73) | (0.69,0.74) |
| p-values | 0.128 |  |  |  |  | 0.238 | 0.716 |  |  |  |  | 0.301 |
| Flucloxacillin | | | | | | | | | | | | |
| Eligible | 0.26 | 0.22 | 0.22 | 0.30 | 0.27 | 0.25 | 0.22 | 0.25 | 0.23 | 0.16 | 0.15 | 0.15 |
|  | (0.16,0.37) | (0.11,0.33) | (0.11,0.32) | (0.18,0.42) | (0.16,0.39) | (0.14,0.37) | (0.15,0.3) | (0.17,0.34) | (0.15,0.31) | (0.09,0.23) | (0.08,0.23) | (0.08,0.23) |
| Ineligible | 0.28 | 0.26 | 0.27 | 0.26 | 0.24 | 0.23 | 0.28 | 0.27 | 0.26 | 0.23 | 0.24 | 0.22 |
|  | (0.25,0.31) | (0.23,0.29) | (0.24,0.3) | (0.23,0.29) | (0.21,0.27) | (0.2,0.26) | (0.26,0.3) | (0.25,0.29) | (0.24,0.28) | (0.21,0.25) | (0.22,0.26) | (0.2,0.25) |
| p-values | 0.879 |  |  |  |  | 0.789 | 0.208 |  |  |  |  | 0.161 |
| Supplementary feeding (oral) | | | | | | | | | | | | |
| Eligible | 0.25 | 0.33 | 0.37 | 0.36 | 0.33 | 0.31 | 0.33 | 0.31 | 0.30 | 0.19 | 0.30 | 0.25 |
|  | (0.15,0.35) | (0.21,0.46) | (0.24,0.49) | (0.23,0.48) | (0.2,0.45) | (0.19,0.44) | (0.25,0.42) | (0.22,0.4) | (0.21,0.39) | (0.12,0.27) | (0.21,0.39) | (0.16,0.34) |
| Ineligible | 0.31 | 0.34 | 0.35 | 0.33 | 0.35 | 0.37 | 0.39 | 0.41 | 0.42 | 0.39 | 0.39 | 0.41 |
|  | (0.28,0.34) | (0.31,0.38) | (0.32,0.38) | (0.3,0.37) | (0.31,0.38) | (0.33,0.4) | (0.37,0.41) | (0.39,0.43) | (0.4,0.44) | (0.37,0.41) | (0.36,0.41) | (0.39,0.44) |
| p-values | 0.314 |  |  |  |  | 0.525 | 0.203 |  |  |  |  | 0.003 |
| Supplementary feeding (gastrostomy) | | | | | | | | | | | | |
| Eligible | 0.06 | 0.06 | 0.08 | 0.09 | 0.09 | 0.12 | 0.14 | 0.12 | 0.12 | 0.11 | 0.09 | 0.11 |
|  | (0,0.11) | (0,0.12) | (0.01,0.15) | (0.01,0.16) | (0.01,0.17) | (0.03,0.21) | (0.08,0.21) | (0.06,0.18) | (0.06,0.19) | (0.05,0.17) | (0.04,0.15) | (0.04,0.17) |
| Ineligible | 0.09 | 0.12 | 0.13 | 0.12 | 0.12 | 0.12 | 0.14 | 0.14 | 0.14 | 0.13 | 0.13 | 0.13 |
|  | (0.07,0.11) | (0.09,0.14) | (0.1,0.15) | (0.1,0.14) | (0.1,0.14) | (0.1,0.15) | (0.12,0.15) | (0.13,0.16) | (0.12,0.16) | (0.12,0.15) | (0.11,0.15) | (0.12,0.15) |
| p-values | 0.460 |  |  |  |  | 1 | 0.916 |  |  |  |  | 0.580 |

| Supplementary Table 5: Proportions (and 95% confidence intervals) in each cohort (children only) taking different treatments by year. P-values from hypothesis test of a difference in proportions are also given for the baseline year and final follow-up year in each era. | | | | | | | | | | | | |
| --- | --- | --- | --- | --- | --- | --- | --- | --- | --- | --- | --- | --- |
|  | Pre-ivacaftor era | | | | | | Post-ivacaftor era | | | | | |
|  | 2007 | 2008 | 2009 | 2010 | 2011 | 2012 | 2013 | 2014 | 2015 | 2016 | 2017 | 2018 |
| Inhaled Antibiotics | | | | | | | | | | | | |
| Eligible | 0.48 | 0.55 | 0.60 | 0.58 | 0.58 | 0.69 | 0.59 | 0.54 | 0.48 | 0.40 | 0.32 | 0.30 |
|  | (0.4,0.56) | (0.46,0.64) | (0.52,0.69) | (0.49,0.67) | (0.5,0.67) | (0.61,0.77) | (0.51,0.67) | (0.46,0.61) | (0.41,0.56) | (0.32,0.48) | (0.25,0.39) | (0.23,0.37) |
| Ineligible | 0.53 | 0.61 | 0.61 | 0.66 | 0.66 | 0.70 | 0.59 | 0.62 | 0.62 | 0.55 | 0.56 | 0.57 |
|  | (0.5,0.55) | (0.59,0.64) | (0.59,0.63) | (0.63,0.68) | (0.64,0.69) | (0.68,0.72) | (0.57,0.61) | (0.6,0.64) | (0.61,0.64) | (0.53,0.57) | (0.54,0.58) | (0.55,0.59) |
| p-values | 0.336 |  |  |  |  | 0.843 | 1 |  |  |  |  | <0.001 |
| Dornase alfa | | | | | | | | | | | | |
| Eligible | 0.30 | 0.40 | 0.50 | 0.53 | 0.57 | 0.62 | 0.63 | 0.59 | 0.62 | 0.59 | 0.60 | 0.63 |
|  | (0.22,0.37) | (0.31,0.49) | (0.41,0.59) | (0.44,0.62) | (0.48,0.65) | (0.54,0.71) | (0.55,0.7) | (0.52,0.67) | (0.54,0.7) | (0.51,0.67) | (0.52,0.68) | (0.55,0.7) |
| Ineligible | 0.34 | 0.43 | 0.50 | 0.55 | 0.59 | 0.65 | 0.60 | 0.69 | 0.77 | 0.79 | 0.83 | 0.85 |
|  | (0.31,0.36) | (0.41,0.46) | (0.47,0.52) | (0.53,0.58) | (0.57,0.62) | (0.62,0.67) | (0.58,0.62) | (0.67,0.71) | (0.75,0.78) | (0.78,0.81) | (0.82,0.85) | (0.83,0.86) |
| p-values | 0.375 |  |  |  |  | 0.673 | 0.547 |  |  |  |  | <0.001 |
| Hypertonic saline solution | | | | | | | | | | | | |
| Eligible | 0.03 | 0.04 | 0.05 | 0.11 | 0.14 | 0.24 | 0.28 | 0.28 | 0.25 | 0.25 | 0.25 | 0.21 |
|  | (0,0.06) | (0,0.07) | (0.01,0.08) | (0.06,0.17) | (0.08,0.2) | (0.17,0.31) | (0.21,0.35) | (0.21,0.35) | (0.18,0.32) | (0.18,0.32) | (0.18,0.32) | (0.15,0.28) |
| Ineligible | 0.02 | 0.05 | 0.08 | 0.14 | 0.20 | 0.27 | 0.29 | 0.34 | 0.40 | 0.40 | 0.43 | 0.46 |
|  | (0.02,0.03) | (0.04,0.06) | (0.07,0.1) | (0.12,0.16) | (0.18,0.22) | (0.25,0.3) | (0.27,0.31) | (0.32,0.36) | (0.38,0.41) | (0.38,0.42) | (0.41,0.45) | (0.44,0.48) |
| p-values | 0.528 |  |  |  |  | 0.464 | 0.966 |  |  |  |  | <0.001 |
| Chronic oral antibiotics | | | | | | | | | | | | |
| Eligible | 0.50 | 0.64 | 0.62 | 0.56 | 0.58 | 0.61 | 0.70 | 0.69 | 0.66 | 0.56 | 0.52 | 0.48 |
|  | (0.42,0.58) | (0.55,0.73) | (0.54,0.71) | (0.48,0.65) | (0.49,0.66) | (0.52,0.69) | (0.63,0.78) | (0.61,0.76) | (0.58,0.73) | (0.48,0.64) | (0.44,0.6) | (0.4,0.56) |
| Ineligible | 0.47 | 0.49 | 0.48 | 0.48 | 0.47 | 0.49 | 0.63 | 0.66 | 0.65 | 0.58 | 0.55 | 0.54 |
|  | (0.44,0.49) | (0.47,0.52) | (0.46,0.51) | (0.46,0.51) | (0.44,0.49) | (0.46,0.51) | (0.61,0.65) | (0.64,0.68) | (0.63,0.67) | (0.56,0.6) | (0.53,0.57) | (0.52,0.56) |
| p-values | 0.553 |  |  |  |  | 0.012 | 0.079 |  |  |  |  | 0.211 |
| Azithromycin | | | | | | | | | | | | |
| Eligible | 0.17 | 0.21 | 0.38 | 0.40 | 0.42 | 0.42 | 0.31 | 0.31 | 0.31 | 0.31 | 0.32 | 0.31 |
|  | (0.11,0.23) | (0.14,0.29) | (0.29,0.46) | (0.31,0.48) | (0.34,0.51) | (0.33,0.5) | (0.24,0.39) | (0.23,0.38) | (0.24,0.38) | (0.23,0.38) | (0.25,0.39) | (0.23,0.38) |
| Ineligible | 0.16 | 0.28 | 0.37 | 0.44 | 0.47 | 0.44 | 0.28 | 0.29 | 0.32 | 0.35 | 0.39 | 0.42 |
|  | (0.14,0.17) | (0.25,0.3) | (0.35,0.4) | (0.41,0.46) | (0.45,0.5) | (0.42,0.47) | (0.27,0.3) | (0.27,0.31) | (0.3,0.34) | (0.33,0.36) | (0.37,0.41) | (0.4,0.44) |
| p-values | 0.705 |  |  |  |  | 0.595 | 0.458 |  |  |  |  | 0.011 |
| Flucloxacillin | | | | | | | | | | | | |
| Eligible | 0.39 | 0.44 | 0.42 | 0.40 | 0.39 | 0.39 | 0.53 | 0.51 | 0.49 | 0.39 | 0.36 | 0.33 |
|  | (0.31,0.47) | (0.35,0.53) | (0.34,0.51) | (0.31,0.48) | (0.31,0.48) | (0.31,0.48) | (0.45,0.61) | (0.43,0.59) | (0.41,0.57) | (0.32,0.47) | (0.28,0.44) | (0.26,0.41) |
| Ineligible | 0.31 | 0.34 | 0.34 | 0.34 | 0.32 | 0.31 | 0.45 | 0.45 | 0.44 | 0.36 | 0.34 | 0.32 |
|  | (0.29,0.33) | (0.31,0.36) | (0.31,0.36) | (0.32,0.36) | (0.29,0.34) | (0.29,0.33) | (0.43,0.47) | (0.43,0.47) | (0.42,0.46) | (0.35,0.38) | (0.32,0.36) | (0.3,0.34) |
| p-values | 0.079 |  |  |  |  | 0.080 | 0.063 |  |  |  |  | 0.814 |
| Supplementary feeding (oral) | | | | | | | | | | | | |
| Eligible | 0.17 | 0.16 | 0.27 | 0.25 | 0.25 | 0.30 | 0.19 | 0.13 | 0.21 | 0.17 | 0.21 | 0.22 |
|  | (0.11,0.23) | (0.09,0.23) | (0.19,0.34) | (0.18,0.33) | (0.18,0.33) | (0.22,0.38) | (0.13,0.26) | (0.08,0.18) | (0.15,0.28) | (0.11,0.23) | (0.14,0.27) | (0.15,0.29) |
| Ineligible | 0.16 | 0.22 | 0.25 | 0.26 | 0.24 | 0.25 | 0.25 | 0.27 | 0.29 | 0.31 | 0.31 | 0.32 |
|  | (0.14,0.17) | (0.2,0.25) | (0.23,0.27) | (0.24,0.28) | (0.22,0.27) | (0.23,0.28) | (0.23,0.27) | (0.25,0.28) | (0.27,0.31) | (0.29,0.32) | (0.29,0.32) | (0.3,0.34) |
| p-values | 0.887 |  |  |  |  | 0.351 | 0.135 |  |  |  |  | 0.011 |
| Supplementary feeding (gastrostomy) | | | | | | | | | | | | |
| Eligible | 0.01 | 0.04 | 0.06 | 0.04 | 0.07 | 0.10 | 0.09 | 0.05 | 0.05 | 0.03 | 0.05 | 0.05 |
|  | (0,0.03) | (0,0.07) | (0.02,0.1) | (0.01,0.07) | (0.03,0.11) | (0.04,0.15) | (0.05,0.14) | (0.02,0.09) | (0.02,0.09) | (0,0.06) | (0.02,0.09) | (0.02,0.09) |
| Ineligible | 0.05 | 0.08 | 0.09 | 0.10 | 0.09 | 0.09 | 0.07 | 0.08 | 0.09 | 0.09 | 0.09 | 0.09 |
|  | (0.04,0.06) | (0.07,0.1) | (0.07,0.1) | (0.08,0.11) | (0.08,0.11) | (0.08,0.11) | (0.06,0.08) | (0.07,0.09) | (0.07,0.1) | (0.08,0.1) | (0.07,0.1) | (0.08,0.1) |
| p-values | 0.087 |  |  |  |  | 1 | 0.384 |  |  |  |  | 0.175 |

| Supplementary Table 6: Proportions (and 95% confidence intervals) in each cohort (adults only) taking different treatments by year. P-values from hypothesis test of a difference in proportions are also given for the baseline year and final follow-up year in each era. | | | | | | | | | | | | |
| --- | --- | --- | --- | --- | --- | --- | --- | --- | --- | --- | --- | --- |
|  | Pre-ivacaftor era | | | | | | Post-ivacaftor era | | | | | |
|  | 2007 | 2008 | 2009 | 2010 | 2011 | 2012 | 2013 | 2014 | 2015 | 2016 | 2017 | 2018 |
| Inhaled Antibiotics | | | | | | | | | | | | |
| Eligible | 0.42 | 0.52 | 0.49 | 0.57 | 0.63 | 0.63 | 0.68 | 0.68 | 0.65 | 0.55 | 0.51 | 0.47 |
|  | (0.34,0.5) | (0.44,0.6) | (0.42,0.55) | (0.51,0.63) | (0.57,0.69) | (0.56,0.69) | (0.62,0.74) | (0.62,0.74) | (0.59,0.71) | (0.49,0.62) | (0.44,0.57) | (0.4,0.54) |
| Ineligible | 0.51 | 0.58 | 0.55 | 0.60 | 0.62 | 0.64 | 0.69 | 0.70 | 0.66 | 0.57 | 0.57 | 0.56 |
|  | (0.49,0.53) | (0.57,0.6) | (0.54,0.57) | (0.58,0.61) | (0.61,0.64) | (0.62,0.65) | (0.67,0.7) | (0.68,0.71) | (0.64,0.67) | (0.55,0.58) | (0.55,0.58) | (0.54,0.58) |
| p-values | 0.046 |  |  |  |  | 0.713 | 0.906 |  |  |  |  | 0.013 |
| Dornase alfa | | | | | | | | | | | | |
| Eligible | 0.30 | 0.40 | 0.38 | 0.51 | 0.46 | 0.49 | 0.58 | 0.59 | 0.59 | 0.56 | 0.53 | 0.54 |
|  | (0.22,0.37) | (0.32,0.48) | (0.31,0.44) | (0.45,0.58) | (0.4,0.52) | (0.42,0.55) | (0.51,0.64) | (0.53,0.65) | (0.53,0.66) | (0.5,0.62) | (0.46,0.59) | (0.47,0.6) |
| Ineligible | 0.36 | 0.45 | 0.46 | 0.52 | 0.54 | 0.58 | 0.63 | 0.65 | 0.66 | 0.65 | 0.68 | 0.70 |
|  | (0.33,0.38) | (0.44,0.47) | (0.44,0.48) | (0.5,0.54) | (0.53,0.56) | (0.57,0.6) | (0.61,0.64) | (0.64,0.67) | (0.65,0.68) | (0.63,0.66) | (0.67,0.7) | (0.69,0.72) |
| p-values | 0.180 |  |  |  |  | 0.005 | 0.131 |  |  |  |  | <0.001 |
| Hypertonic saline solution | | | | | | | | | | | | |
| Eligible | 0.03 | 0.06 | 0.06 | 0.13 | 0.18 | 0.25 | 0.30 | 0.31 | 0.30 | 0.29 | 0.28 | 0.26 |
|  | (0,0.06) | (0.02,0.09) | (0.03,0.09) | (0.09,0.18) | (0.13,0.23) | (0.2,0.31) | (0.24,0.36) | (0.25,0.37) | (0.24,0.35) | (0.23,0.35) | (0.22,0.33) | (0.2,0.32) |
| Ineligible | 0.07 | 0.08 | 0.09 | 0.15 | 0.21 | 0.25 | 0.29 | 0.34 | 0.34 | 0.31 | 0.33 | 0.34 |
|  | (0.06,0.08) | (0.07,0.09) | (0.08,0.1) | (0.14,0.17) | (0.19,0.22) | (0.23,0.26) | (0.28,0.31) | (0.32,0.35) | (0.33,0.36) | (0.3,0.33) | (0.32,0.35) | (0.33,0.36) |
| p-values | 0.079 |  |  |  |  | 0.953 | 0.900 |  |  |  |  | 0.013 |
| Chronic oral antibiotics | | | | | | | | | | | | |
| Eligible | 0.30 | 0.33 | 0.33 | 0.37 | 0.36 | 0.34 | 0.34 | 0.39 | 0.36 | 0.29 | 0.29 | 0.29 |
|  | (0.23,0.38) | (0.26,0.4) | (0.27,0.39) | (0.31,0.44) | (0.3,0.42) | (0.28,0.4) | (0.28,0.4) | (0.32,0.45) | (0.3,0.43) | (0.23,0.35) | (0.23,0.35) | (0.23,0.35) |
| Ineligible | 0.37 | 0.38 | 0.41 | 0.41 | 0.41 | 0.43 | 0.41 | 0.45 | 0.44 | 0.43 | 0.41 | 0.39 |
|  | (0.35,0.39) | (0.36,0.4) | (0.39,0.42) | (0.4,0.43) | (0.39,0.42) | (0.41,0.44) | (0.39,0.42) | (0.44,0.47) | (0.43,0.46) | (0.41,0.44) | (0.39,0.42) | (0.38,0.41) |
| p-values | 0.122 |  |  |  |  | 0.012 | 0.054 |  |  |  |  | 0.004 |
| Azithromycin | | | | | | | | | | | | |
| Eligible | 0.36 | 0.49 | 0.47 | 0.57 | 0.60 | 0.62 | 0.62 | 0.56 | 0.60 | 0.52 | 0.56 | 0.57 |
|  | (0.28,0.44) | (0.41,0.56) | (0.4,0.53) | (0.51,0.64) | (0.54,0.66) | (0.56,0.68) | (0.56,0.68) | (0.5,0.62) | (0.53,0.66) | (0.46,0.59) | (0.49,0.62) | (0.5,0.64) |
| Ineligible | 0.38 | 0.48 | 0.46 | 0.55 | 0.57 | 0.54 | 0.61 | 0.59 | 0.58 | 0.57 | 0.62 | 0.62 |
|  | (0.36,0.4) | (0.47,0.5) | (0.45,0.48) | (0.53,0.56) | (0.55,0.59) | (0.53,0.56) | (0.59,0.62) | (0.58,0.61) | (0.56,0.59) | (0.56,0.59) | (0.6,0.63) | (0.6,0.63) |
| p-values | 0.745 |  |  |  |  | 0.020 | 0.715 |  |  |  |  | 0.216 |
| Flucloxacillin | | | | | | | | | | | | |
| Eligible | 0.18 | 0.24 | 0.23 | 0.27 | 0.23 | 0.19 | 0.21 | 0.24 | 0.23 | 0.19 | 0.20 | 0.18 |
|  | (0.12,0.25) | (0.17,0.3) | (0.17,0.28) | (0.21,0.33) | (0.18,0.29) | (0.14,0.24) | (0.15,0.26) | (0.19,0.29) | (0.17,0.28) | (0.14,0.24) | (0.14,0.25) | (0.13,0.24) |
| Ineligible | 0.26 | 0.28 | 0.29 | 0.30 | 0.30 | 0.29 | 0.26 | 0.28 | 0.27 | 0.25 | 0.26 | 0.24 |
|  | (0.24,0.27) | (0.26,0.3) | (0.27,0.3) | (0.29,0.32) | (0.29,0.31) | (0.27,0.3) | (0.25,0.28) | (0.26,0.29) | (0.26,0.28) | (0.24,0.27) | (0.25,0.27) | (0.23,0.26) |
| p-values | 0.065 |  |  |  |  | 0.002 | 0.050 |  |  |  |  | 0.057 |
| Supplementary feeding (oral) | | | | | | | | | | | | |
| Eligible | 0.16 | 0.22 | 0.25 | 0.24 | 0.25 | 0.25 | 0.25 | 0.24 | 0.24 | 0.18 | 0.19 | 0.21 |
|  | (0.1,0.22) | (0.16,0.29) | (0.19,0.31) | (0.18,0.29) | (0.2,0.31) | (0.2,0.31) | (0.19,0.3) | (0.19,0.29) | (0.18,0.29) | (0.13,0.23) | (0.14,0.24) | (0.16,0.27) |
| Ineligible | 0.19 | 0.27 | 0.26 | 0.25 | 0.28 | 0.29 | 0.30 | 0.31 | 0.32 | 0.29 | 0.28 | 0.30 |
|  | (0.18,0.21) | (0.25,0.29) | (0.25,0.28) | (0.24,0.27) | (0.27,0.29) | (0.27,0.3) | (0.29,0.31) | (0.3,0.33) | (0.3,0.33) | (0.28,0.31) | (0.27,0.29) | (0.28,0.31) |
| p-values | 0.397 |  |  |  |  | 0.242 | 0.089 |  |  |  |  | 0.013 |
| Supplementary feeding (gastrostomy) | | | | | | | | | | | | |
| Eligible | 0.04 | 0.04 | 0.06 | 0.07 | 0.06 | 0.06 | 0.05 | 0.05 | 0.04 | 0.05 | 0.02 | 0.02 |
|  | (0,0.07) | (0.01,0.07) | (0.03,0.09) | (0.03,0.1) | (0.03,0.09) | (0.03,0.09) | (0.03,0.08) | (0.02,0.07) | (0.01,0.06) | (0.02,0.08) | (0,0.04) | (0,0.04) |
| Ineligible | 0.03 | 0.06 | 0.06 | 0.06 | 0.07 | 0.06 | 0.07 | 0.06 | 0.06 | 0.05 | 0.05 | 0.05 |
|  | (0.03,0.04) | (0.05,0.07) | (0.05,0.07) | (0.06,0.07) | (0.06,0.07) | (0.06,0.07) | (0.06,0.07) | (0.06,0.07) | (0.05,0.06) | (0.04,0.06) | (0.04,0.06) | (0.04,0.06) |
| p-values | 1 |  |  |  |  | 0.848 | 0.517 |  |  |  |  | 0.057 |

| Supplementary Table 7: Proportions (and 95% confidence intervals) in each cohort (females only) taking different treatments by year. P-values from hypothesis test of a difference in proportions are also given for the baseline year and final follow-up year in each era. | | | | | | | | | | | | |
| --- | --- | --- | --- | --- | --- | --- | --- | --- | --- | --- | --- | --- |
|  | Pre-ivacaftor era | | | | | | Post-ivacaftor era | | | | | |
|  | 2007 | 2008 | 2009 | 2010 | 2011 | 2012 | 2013 | 2014 | 2015 | 2016 | 2017 | 2018 |
| Inhaled Antibiotics | | | | | | | | | | | | |
| Eligible | 0.46 | 0.57 | 0.56 | 0.58 | 0.62 | 0.66 | 0.65 | 0.64 | 0.55 | 0.51 | 0.46 | 0.42 |
|  | (0.37,0.54) | (0.48,0.65) | (0.48,0.63) | (0.5,0.65) | (0.54,0.69) | (0.59,0.73) | (0.58,0.72) | (0.58,0.71) | (0.48,0.62) | (0.43,0.58) | (0.38,0.53) | (0.34,0.49) |
| Ineligible | 0.51 | 0.60 | 0.57 | 0.61 | 0.63 | 0.66 | 0.65 | 0.67 | 0.66 | 0.58 | 0.58 | 0.58 |
|  | (0.49,0.53) | (0.58,0.62) | (0.55,0.59) | (0.59,0.63) | (0.61,0.65) | (0.64,0.68) | (0.64,0.67) | (0.66,0.69) | (0.64,0.68) | (0.56,0.59) | (0.56,0.6) | (0.56,0.6) |
| p-values | 0.243 |  |  |  |  | 1 | 0.980 |  |  |  |  | <0.001 |
| Dornase alfa | | | | | | | | | | | | |
| Eligible | 0.29 | 0.41 | 0.45 | 0.53 | 0.52 | 0.56 | 0.61 | 0.62 | 0.63 | 0.59 | 0.59 | 0.63 |
|  | (0.21,0.37) | (0.33,0.49) | (0.37,0.53) | (0.45,0.6) | (0.45,0.59) | (0.49,0.64) | (0.54,0.68) | (0.55,0.69) | (0.56,0.7) | (0.52,0.66) | (0.52,0.67) | (0.56,0.7) |
| Ineligible | 0.37 | 0.46 | 0.49 | 0.55 | 0.57 | 0.61 | 0.63 | 0.68 | 0.72 | 0.72 | 0.76 | 0.77 |
|  | (0.34,0.39) | (0.44,0.49) | (0.47,0.51) | (0.53,0.57) | (0.55,0.59) | (0.59,0.63) | (0.61,0.65) | (0.67,0.7) | (0.7,0.74) | (0.7,0.73) | (0.74,0.77) | (0.76,0.79) |
| p-values | 0.098 |  |  |  |  | 0.246 | 0.614 |  |  |  |  | <0.001 |
| Hypertonic saline solution | | | | | | | | | | | | |
| Eligible | 0.04 | 0.07 | 0.08 | 0.13 | 0.17 | 0.27 | 0.29 | 0.31 | 0.29 | 0.27 | 0.27 | 0.24 |
|  | (0.01,0.08) | (0.02,0.11) | (0.04,0.12) | (0.08,0.18) | (0.11,0.22) | (0.2,0.34) | (0.22,0.35) | (0.25,0.38) | (0.22,0.35) | (0.21,0.34) | (0.2,0.33) | (0.18,0.31) |
| Ineligible | 0.05 | 0.07 | 0.10 | 0.16 | 0.23 | 0.29 | 0.32 | 0.37 | 0.39 | 0.37 | 0.40 | 0.42 |
|  | (0.04,0.06) | (0.06,0.08) | (0.09,0.11) | (0.14,0.17) | (0.21,0.24) | (0.27,0.31) | (0.31,0.34) | (0.35,0.39) | (0.38,0.41) | (0.36,0.39) | (0.38,0.42) | (0.4,0.43) |
| p-values | 0.786 |  |  |  |  | 0.682 | 0.343 |  |  |  |  | <0.001 |
| Chronic oral antibiotics | | | | | | | | | | | | |
| Eligible | 0.40 | 0.40 | 0.38 | 0.40 | 0.37 | 0.36 | 0.44 | 0.48 | 0.47 | 0.43 | 0.43 | 0.42 |
|  | (0.32,0.49) | (0.32,0.49) | (0.3,0.45) | (0.33,0.47) | (0.3,0.44) | (0.29,0.44) | (0.37,0.51) | (0.41,0.55) | (0.39,0.54) | (0.36,0.51) | (0.36,0.5) | (0.35,0.5) |
| Ineligible | 0.40 | 0.41 | 0.42 | 0.42 | 0.41 | 0.42 | 0.48 | 0.52 | 0.50 | 0.47 | 0.45 | 0.44 |
|  | (0.37,0.42) | (0.39,0.43) | (0.4,0.44) | (0.4,0.44) | (0.39,0.43) | (0.4,0.44) | (0.46,0.5) | (0.5,0.53) | (0.49,0.52) | (0.45,0.49) | (0.43,0.47) | (0.42,0.45) |
| p-values | 0.941 |  |  |  |  | 0.155 | 0.255 |  |  |  |  | 0.800 |
| Azithromycin | | | | | | | | | | | | |
| Eligible | 0.28 | 0.38 | 0.45 | 0.48 | 0.51 | 0.51 | 0.47 | 0.41 | 0.44 | 0.40 | 0.42 | 0.39 |
|  | (0.2,0.35) | (0.3,0.46) | (0.37,0.53) | (0.41,0.56) | (0.44,0.59) | (0.44,0.59) | (0.4,0.54) | (0.34,0.49) | (0.37,0.52) | (0.33,0.47) | (0.35,0.5) | (0.31,0.46) |
| Ineligible | 0.29 | 0.43 | 0.46 | 0.52 | 0.54 | 0.52 | 0.48 | 0.48 | 0.48 | 0.49 | 0.53 | 0.54 |
|  | (0.27,0.31) | (0.4,0.45) | (0.44,0.48) | (0.5,0.54) | (0.52,0.56) | (0.5,0.54) | (0.46,0.5) | (0.46,0.5) | (0.46,0.49) | (0.47,0.5) | (0.51,0.55) | (0.52,0.56) |
| p-values | 0.755 |  |  |  |  | 0.818 | 0.848 |  |  |  |  | <0.001 |
| Flucloxacillin | | | | | | | | | | | | |
| Eligible | 0.31 | 0.32 | 0.28 | 0.31 | 0.27 | 0.24 | 0.32 | 0.37 | 0.36 | 0.32 | 0.31 | 0.31 |
|  | (0.23,0.39) | (0.24,0.4) | (0.21,0.35) | (0.24,0.38) | (0.2,0.33) | (0.17,0.3) | (0.25,0.38) | (0.3,0.44) | (0.29,0.43) | (0.25,0.39) | (0.25,0.38) | (0.24,0.38) |
| Ineligible | 0.27 | 0.29 | 0.30 | 0.30 | 0.28 | 0.28 | 0.33 | 0.33 | 0.32 | 0.28 | 0.28 | 0.26 |
|  | (0.25,0.29) | (0.27,0.31) | (0.28,0.32) | (0.28,0.32) | (0.27,0.3) | (0.27,0.3) | (0.31,0.34) | (0.31,0.34) | (0.3,0.34) | (0.27,0.3) | (0.26,0.29) | (0.24,0.28) |
| p-values | 0.371 |  |  |  |  | 0.213 | 0.880 |  |  |  |  | 0.176 |
| Supplementary feeding (oral) | | | | | | | | | | | | |
| Eligible | 0.14 | 0.16 | 0.22 | 0.19 | 0.18 | 0.19 | 0.16 | 0.14 | 0.19 | 0.17 | 0.17 | 0.19 |
|  | (0.08,0.2) | (0.1,0.22) | (0.16,0.29) | (0.13,0.24) | (0.12,0.24) | (0.13,0.25) | (0.11,0.21) | (0.09,0.19) | (0.14,0.25) | (0.12,0.22) | (0.11,0.22) | (0.13,0.25) |
| Ineligible | 0.15 | 0.21 | 0.23 | 0.23 | 0.23 | 0.23 | 0.25 | 0.26 | 0.27 | 0.26 | 0.25 | 0.28 |
|  | (0.14,0.17) | (0.2,0.23) | (0.21,0.24) | (0.21,0.25) | (0.21,0.24) | (0.22,0.25) | (0.23,0.26) | (0.24,0.27) | (0.25,0.28) | (0.24,0.28) | (0.23,0.27) | (0.26,0.29) |
| p-values | 0.779 |  |  |  |  | 0.211 | 0.009 |  |  |  |  | 0.020 |
| Supplementary feeding (gastrostomy) | | | | | | | | | | | | |
| Eligible | 0.02 | 0.04 | 0.06 | 0.05 | 0.06 | 0.06 | 0.07 | 0.05 | 0.04 | 0.04 | 0.03 | 0.02 |
|  | (0,0.05) | (0.01,0.08) | (0.02,0.1) | (0.02,0.08) | (0.03,0.1) | (0.02,0.09) | (0.03,0.1) | (0.02,0.09) | (0.01,0.06) | (0.01,0.07) | (0.01,0.06) | (0,0.05) |
| Ineligible | 0.04 | 0.07 | 0.08 | 0.08 | 0.08 | 0.08 | 0.07 | 0.08 | 0.07 | 0.07 | 0.07 | 0.07 |
|  | (0.03,0.05) | (0.06,0.08) | (0.07,0.09) | (0.07,0.09) | (0.07,0.09) | (0.07,0.09) | (0.06,0.08) | (0.07,0.09) | (0.06,0.08) | (0.06,0.08) | (0.06,0.08) | (0.06,0.08) |
| p-values | 0.412 |  |  |  |  | 0.411 | 1 |  |  |  |  | 0.032 |

| Supplementary Table 8: Proportions (and 95% confidence intervals) in each cohort (males only) taking different treatments by year. P-values from hypothesis test of a difference in proportions are also given for the baseline year and final follow-up year in each era. | | | | | | | | | | | | |
| --- | --- | --- | --- | --- | --- | --- | --- | --- | --- | --- | --- | --- |
|  | Pre-ivacaftor era | | | | | | Post-ivacaftor era | | | | | |
|  | 2007 | 2008 | 2009 | 2010 | 2011 | 2012 | 2013 | 2014 | 2015 | 2016 | 2017 | 2018 |
| Inhaled Antibiotics | | | | | | | | | | | | |
| Eligible | 0.45 | 0.50 | 0.51 | 0.57 | 0.61 | 0.64 | 0.64 | 0.60 | 0.61 | 0.48 | 0.41 | 0.38 |
|  | (0.37,0.53) | (0.41,0.58) | (0.43,0.58) | (0.5,0.64) | (0.54,0.68) | (0.57,0.71) | (0.58,0.71) | (0.54,0.67) | (0.54,0.68) | (0.41,0.55) | (0.34,0.48) | (0.31,0.45) |
| Ineligible | 0.53 | 0.59 | 0.57 | 0.61 | 0.63 | 0.65 | 0.65 | 0.66 | 0.63 | 0.55 | 0.55 | 0.55 |
|  | (0.5,0.55) | (0.57,0.61) | (0.56,0.59) | (0.6,0.63) | (0.61,0.65) | (0.64,0.67) | (0.63,0.66) | (0.65,0.68) | (0.62,0.65) | (0.53,0.56) | (0.54,0.57) | (0.53,0.56) |
| p-values | 0.088 |  |  |  |  | 0.693 | 1 |  |  |  |  | <0.001 |
| Dornase alfa | | | | | | | | | | | | |
| Eligible | 0.30 | 0.39 | 0.40 | 0.51 | 0.48 | 0.51 | 0.59 | 0.56 | 0.58 | 0.56 | 0.52 | 0.52 |
|  | (0.23,0.37) | (0.31,0.47) | (0.32,0.47) | (0.44,0.58) | (0.41,0.55) | (0.43,0.58) | (0.52,0.65) | (0.5,0.63) | (0.51,0.65) | (0.49,0.62) | (0.46,0.59) | (0.45,0.6) |
| Ineligible | 0.33 | 0.43 | 0.46 | 0.52 | 0.55 | 0.59 | 0.60 | 0.65 | 0.69 | 0.69 | 0.72 | 0.75 |
|  | (0.31,0.35) | (0.41,0.45) | (0.44,0.48) | (0.5,0.53) | (0.53,0.57) | (0.57,0.61) | (0.59,0.62) | (0.64,0.67) | (0.67,0.7) | (0.67,0.7) | (0.71,0.74) | (0.73,0.76) |
| p-values | 0.501 |  |  |  |  | 0.029 | 0.637 |  |  |  |  | <0.001 |
| Hypertonic saline solution | | | | | | | | | | | | |
| Eligible | 0.02 | 0.03 | 0.03 | 0.12 | 0.16 | 0.23 | 0.30 | 0.29 | 0.27 | 0.27 | 0.26 | 0.23 |
|  | (0,0.04) | (0,0.06) | (0.01,0.06) | (0.07,0.17) | (0.11,0.21) | (0.17,0.29) | (0.24,0.36) | (0.22,0.35) | (0.21,0.33) | (0.21,0.33) | (0.2,0.32) | (0.17,0.29) |
| Ineligible | 0.05 | 0.07 | 0.08 | 0.14 | 0.19 | 0.23 | 0.26 | 0.31 | 0.34 | 0.33 | 0.35 | 0.37 |
|  | (0.04,0.05) | (0.06,0.08) | (0.07,0.09) | (0.13,0.16) | (0.17,0.2) | (0.21,0.24) | (0.25,0.28) | (0.29,0.32) | (0.32,0.35) | (0.31,0.34) | (0.33,0.37) | (0.35,0.38) |
| p-values | 0.197 |  |  |  |  | 1 | 0.301 |  |  |  |  | <0.001 |
| Chronic oral antibiotics | | | | | | | | | | | | |
| Eligible | 0.40 | 0.51 | 0.49 | 0.48 | 0.49 | 0.49 | 0.53 | 0.52 | 0.50 | 0.36 | 0.34 | 0.32 |
|  | (0.32,0.48) | (0.43,0.59) | (0.42,0.57) | (0.41,0.55) | (0.42,0.56) | (0.42,0.57) | (0.46,0.6) | (0.46,0.59) | (0.43,0.57) | (0.29,0.43) | (0.27,0.41) | (0.26,0.39) |
| Ineligible | 0.43 | 0.43 | 0.44 | 0.45 | 0.44 | 0.46 | 0.51 | 0.55 | 0.54 | 0.50 | 0.47 | 0.46 |
|  | (0.41,0.45) | (0.41,0.45) | (0.42,0.46) | (0.43,0.47) | (0.42,0.46) | (0.44,0.48) | (0.49,0.53) | (0.53,0.56) | (0.52,0.55) | (0.48,0.51) | (0.45,0.49) | (0.44,0.48) |
| p-values | 0.540 |  |  |  |  | 0.396 | 0.647 |  |  |  |  | <0.001 |
| Azithromycin | | | | | | | | | | | | |
| Eligible | 0.25 | 0.37 | 0.42 | 0.54 | 0.56 | 0.58 | 0.53 | 0.50 | 0.52 | 0.47 | 0.50 | 0.52 |
|  | (0.19,0.32) | (0.29,0.45) | (0.35,0.49) | (0.47,0.61) | (0.49,0.63) | (0.51,0.65) | (0.46,0.6) | (0.44,0.57) | (0.45,0.59) | (0.4,0.54) | (0.43,0.56) | (0.45,0.6) |
| Ineligible | 0.27 | 0.41 | 0.41 | 0.51 | 0.54 | 0.51 | 0.48 | 0.47 | 0.48 | 0.49 | 0.53 | 0.54 |
|  | (0.25,0.29) | (0.39,0.43) | (0.39,0.43) | (0.49,0.53) | (0.52,0.56) | (0.49,0.52) | (0.46,0.49) | (0.46,0.49) | (0.46,0.5) | (0.47,0.5) | (0.51,0.54) | (0.52,0.55) |
| p-values | 0.749 |  |  |  |  | 0.042 | 0.158 |  |  |  |  | 0.799 |
| Flucloxacillin | | | | | | | | | | | | |
| Eligible | 0.26 | 0.32 | 0.32 | 0.32 | 0.31 | 0.28 | 0.35 | 0.33 | 0.31 | 0.22 | 0.22 | 0.19 |
|  | (0.19,0.33) | (0.25,0.4) | (0.25,0.39) | (0.25,0.39) | (0.25,0.37) | (0.22,0.35) | (0.28,0.41) | (0.26,0.39) | (0.24,0.37) | (0.16,0.28) | (0.16,0.27) | (0.13,0.25) |
| Ineligible | 0.29 | 0.31 | 0.31 | 0.33 | 0.32 | 0.31 | 0.35 | 0.36 | 0.35 | 0.31 | 0.30 | 0.29 |
|  | (0.27,0.31) | (0.29,0.33) | (0.29,0.32) | (0.31,0.34) | (0.31,0.34) | (0.29,0.32) | (0.33,0.37) | (0.34,0.37) | (0.33,0.36) | (0.29,0.32) | (0.29,0.32) | (0.27,0.3) |
| p-values | 0.578 |  |  |  |  | 0.574 | 0.986 |  |  |  |  | 0.005 |
| Supplementary feeding (oral) | | | | | | | | | | | | |
| Eligible | 0.18 | 0.24 | 0.29 | 0.30 | 0.32 | 0.34 | 0.29 | 0.25 | 0.26 | 0.18 | 0.22 | 0.24 |
|  | (0.12,0.24) | (0.17,0.31) | (0.22,0.35) | (0.23,0.36) | (0.25,0.38) | (0.27,0.4) | (0.22,0.35) | (0.19,0.31) | (0.2,0.32) | (0.13,0.23) | (0.16,0.28) | (0.18,0.3) |
| Ineligible | 0.20 | 0.29 | 0.29 | 0.28 | 0.31 | 0.32 | 0.31 | 0.33 | 0.34 | 0.33 | 0.32 | 0.33 |
|  | (0.18,0.22) | (0.27,0.31) | (0.27,0.3) | (0.26,0.3) | (0.29,0.32) | (0.3,0.33) | (0.29,0.32) | (0.31,0.34) | (0.32,0.35) | (0.32,0.35) | (0.31,0.34) | (0.32,0.35) |
| p-values | 0.728 |  |  |  |  | 0.638 | 0.515 |  |  |  |  | 0.009 |
| Supplementary feeding (gastrostomy) | | | | | | | | | | | | |
| Eligible | 0.03 | 0.03 | 0.05 | 0.07 | 0.07 | 0.08 | 0.07 | 0.04 | 0.05 | 0.05 | 0.04 | 0.04 |
|  | (0,0.05) | (0,0.06) | (0.02,0.09) | (0.03,0.1) | (0.03,0.1) | (0.04,0.12) | (0.04,0.11) | (0.02,0.07) | (0.02,0.08) | (0.02,0.07) | (0.01,0.06) | (0.01,0.07) |
| Ineligible | 0.04 | 0.06 | 0.06 | 0.07 | 0.07 | 0.07 | 0.07 | 0.06 | 0.06 | 0.06 | 0.06 | 0.06 |
|  | (0.03,0.05) | (0.05,0.07) | (0.05,0.07) | (0.06,0.08) | (0.06,0.08) | (0.06,0.08) | (0.06,0.08) | (0.06,0.07) | (0.06,0.07) | (0.05,0.06) | (0.05,0.07) | (0.05,0.07) |
| p-values | 0.510 |  |  |  |  | 0.440 | 0.942 |  |  |  |  | 0.318 |

Supplementary Figure 2: The proportions and 95% confidence intervals in each cohort prescribed different treatments by year, stratified by FEV1% at baseline.


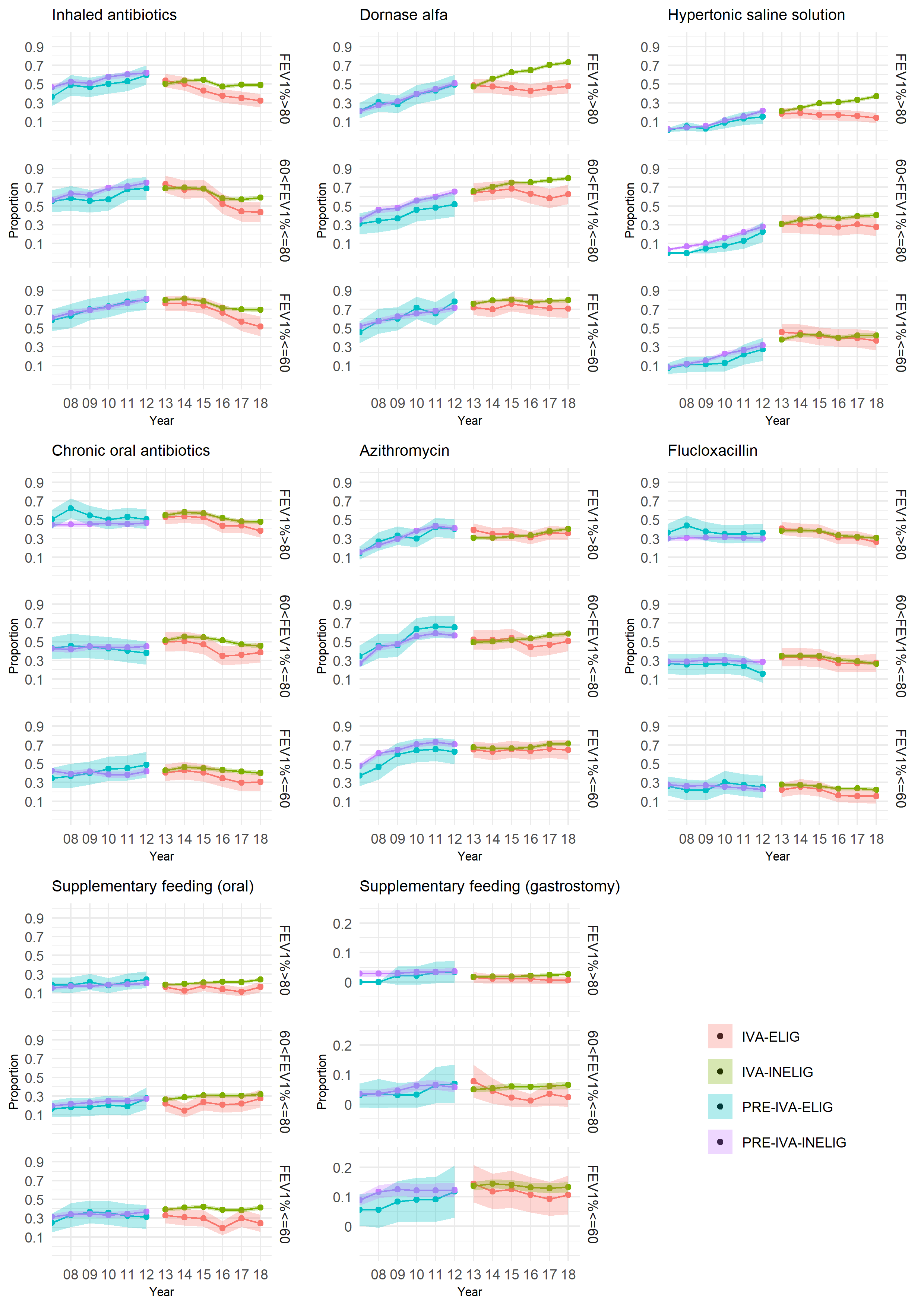


Supplementary Figure 3: The proportions and 95% confidence intervals in each cohort prescribed different treatments by year, stratified by sex.


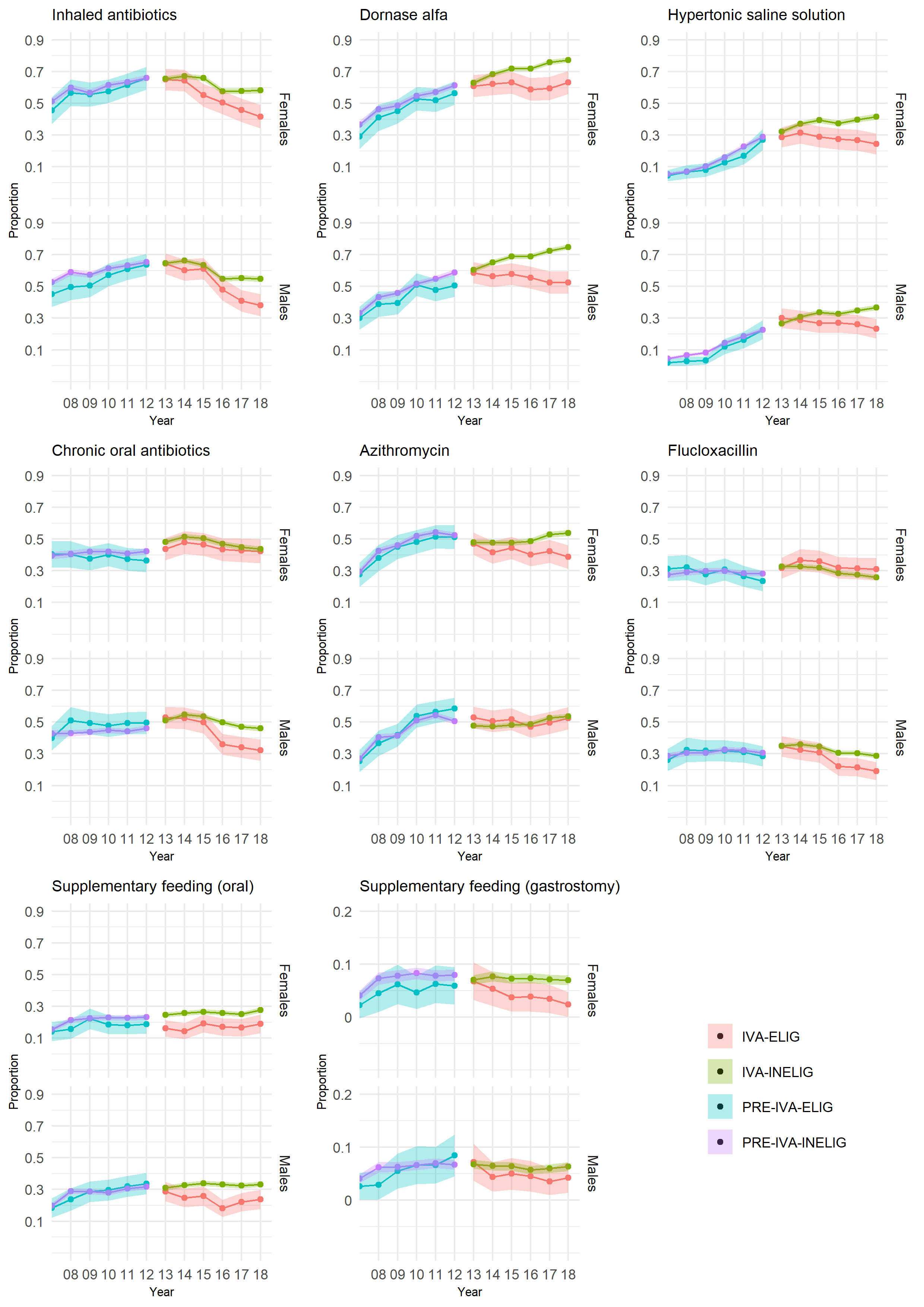


Supplementary Figure 4: The proportions and 95% confidence intervals in each cohort prescribed different treatments by year, stratified by baseline age.


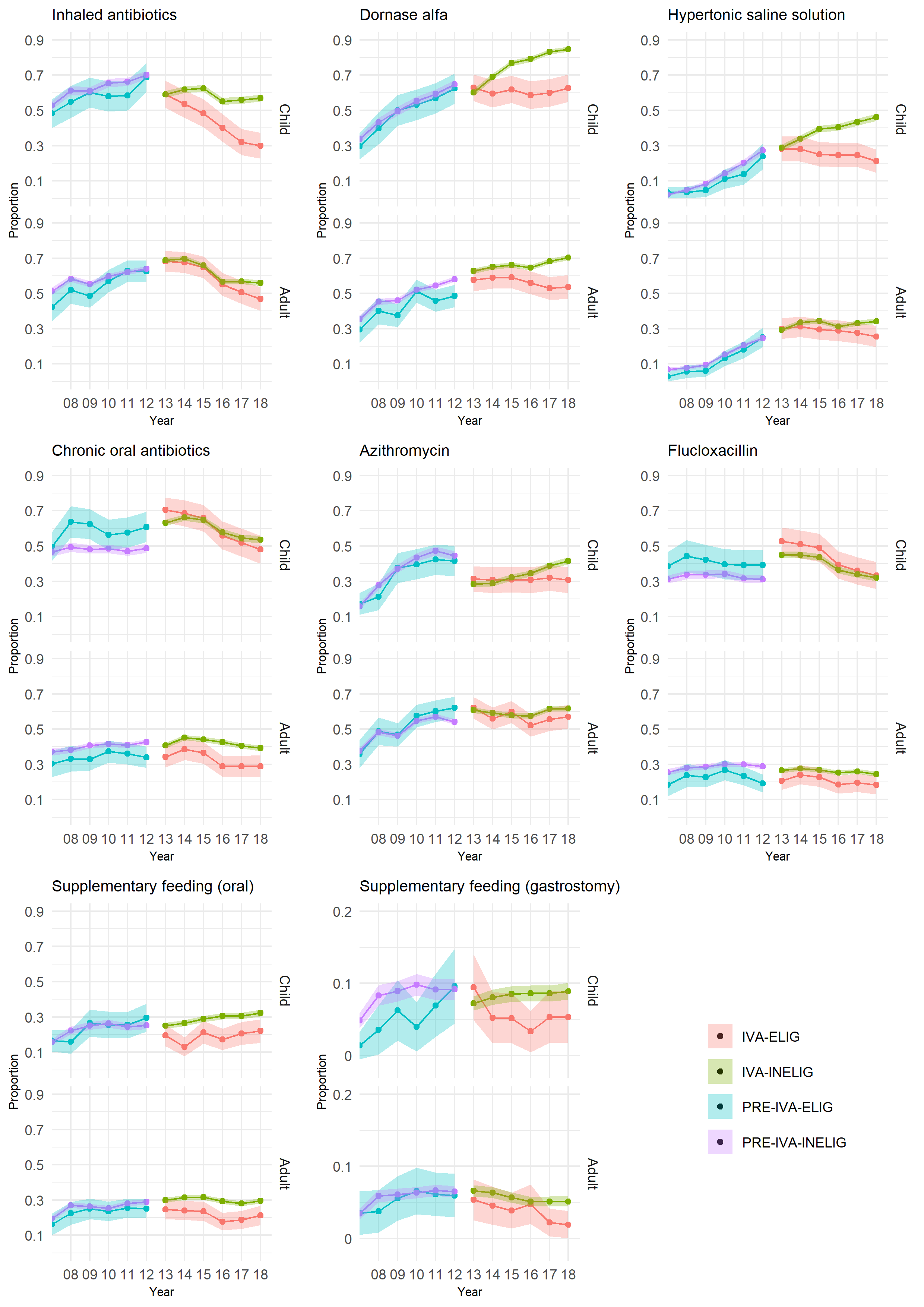


| Supplementary Table 9: P-values associated with hypothesis tests for a difference in the trend of proportions of treatment use over time by genotype group. | | |
| --- | --- | --- |
| Treatment | Pre-ivacaftor era  (2007-2012) | Ivacaftor era  (2013-2018) |
| Inhaled antibiotics | 0.093 | <0.001 |
| Dornase alfa | 0.798 | <0.001 |
| Hypertonic saline solution | 0.066 | <0.001 |
| Chronic oral antibiotics | 0.721 | 0.003 |
| Azithromycin | 0.080 | 0.001 |
| Flucloxacillin | 0.160 | 0.225 |
| Supplementary feeding (oral) | 0.432 | 0.245 |
| Supplementary feeding (gastrostomy) | 0.100 | 0.027 |
